# An agent-based model to investigate the effects of urban segregation around the clock on inequalities in health behaviour

**DOI:** 10.1140/epjds/s13688-025-00603-4

**Published:** 2025-12-11

**Authors:** Clémentine Cottineau-Mugadza, Julien Perret, Romain Reuillon, Sébastien Rey-Coyrehourcq, Julie Vallée

**Affiliations:** 1 UMR 8097 Centre Maurice Halbwachs, CNRS, Paris, France; 2 Delft University of Technology ABE, Delft, The Netherlands; 3 Univ Gustave Eiffel, ENSG, IGN, LASTIG, Champs-sur-Marne, France; 4 Institut des Systèmes Complexes Paris Ile-de-France, Paris, France; 5 UMR 8504 Géographie-cités, CNRS, Paris, France; 6 UMR 6266 IDEES, Université de Rouen, Rouen, France; 7 UMR 5193 LISST, CNRS, Toulouse, France

**Keywords:** Spatial Segregation, Daily Mobility, Social Inequalities, Dietary behaviour, ABM, Neighbourhood effects, Contextual effects

## Abstract

Social segregation in cities refers to the uneven spatial distribution of individuals from unequal social groups, such as affluent and economically vulnerable people. Social segregation may, in turn, produce social inequalities through contextual effects, since neighbourhood mixing or concentration plays a role in shaping individuals’ opinions and behaviours in multiple life domains, including health. Because segregation and contextual effects occur at the places of residence as well as throughout the day, as people move between locations in a city, we aim to understand the social effect of urban segregation ‘around the clock’ on health behaviours (such as the choice of a healthy diet), using an empirical agent-based model initialised on the Paris region with a synthetic population. We built this synthetic population by pulling together data from two health & nutrition surveys conducted 6 years apart, data from the French census and data from an origin-destination survey. We then combined scenarios of residential patterns (random allocation vs. census-based allocation reflecting the empirical level of residential segregation) with scenarios of daily mobility (no daily moves, random moves or survey-based daily moves reflecting the empirical level of daytime segregation in Paris) to assess the effect of spatio-temporal segregation on the diffusion of health behaviours. While the same upward trend of healthy behaviours is obtained in all scenarios simulated, we find contrasted results with respect to social inequalities: 1/ when the agents’ residence is allocated at random, social inequalities of health decrease in the long run; 2/ randomizing daily mobility can mitigate the increase in social inequalities in dietary behaviours induced by effective residential segregation, with this mitigation effect appearing as soon as a small proportion of daily moves are random; 3/ daytime segregation as it exists in Paris slightly reinforces the unequal distribution of health behaviours between the most and least educated groups compared with the sole effect of residential segregation.

**Supplementary Information:**

The online version contains supplementary material available at 10.1140/epjds/s13688-025-00603-4.

## Introduction

Social segregation in cities reflects the fact that people from different social groups tend to locate, attend activities, and travel to and through separated areas, at different times. Segregation thus describes the state of spatial separation of social groups as well as the processes which reinforce it over time, these processes being deliberate or unconscious, chosen or suffered by individuals.

Social segregation stems from two cumulative processes. On the one hand, it is a sorting process whereby people with similar characteristics tend to concentrate in similar places that reflect their preferences, family needs or purchasing power [16, 48, 70]. A classic agent-based model (ABM) that exemplifies the amplifying effects of residential sorting as a social process is that of Schelling [62], in which a slight preference for neighbours similar to oneself at the individual level (e.g. homophily) can produce stark patterns of residential segregation at the city level. However, it has been proven that sorting process and selective migration remain minor drivers of segregation [6], compared to on-site changes such as ageing, social mobility and cohort replacement. As long as residential segregation and people’s home locations do not affect their individual characteristics (opportunities, income, health and educational outcomes) or that of their neighbours, Cheshire et al. [17, p.225] argue that*“policymakers should be more relaxed about residential segregation in cities, but much less relaxed about inequality and poor education”*.

On the other hand, not only are the places one lives in or travels to and through influenced by their individual characteristics, constraints and preferences, but according to the neighbourhood effects literature, spatial contexts influence individual characteristics in return [31, 39, 69, 77]. For example, there is evidence that segregation is particularly detrimental to vulnerable populations (poor people, those with little formal education, single parents, immigrants, those who are socially isolated and, a fortiori, those who combine two or more of these characteristics) facing a doubly unfair situation, especially in the field of health [23]. Spatial contextual effects (or neighbourhood effects) related to social segregation do not only reflect but also add to the individual effects that social status and household income have on individual health levels and behaviours, as underlined by Wilson [80] in his book entitled ‘The truly disadvantaged’. Moreover, neighbourhood effects go beyond neighbourhoods of residence: daily activity locations (work, school, shopping, leisure, etc.) also matter, reflecting the complexity of social interactions of urban dwellers in space and time [73]. However, the accurate representation of these activity spaces and their associated social interactions over the course of the day is usually missing when exploring health inequality dynamics, their interplay and place-based effects.

In this interdisciplinary project, we address the real-world complexity of social geographies when building an agent-based model (ABM) and synthetic population [14, 35] to generate, locate, activate and move several millions of artificial agents in a realistically-sized artificial urban space. Unlike the classic Schelling model of segregation, which produces a stylized pattern of residential segregation in a stylized uniform city of stylized 1-dimensional individuals, our model is calibrated on conventional data sources such as the national census, origin-destination surveys (often more representative and socially precise than digital tracking data) and health surveys to account for more complexity in the mechanisms of neighborhood effects, social segregation and health inequality dynamics. Our synthetic population and ABM can shed new light on the spatiotemporal dynamics of vulnerability and social inequality, with the example of the Paris region in France.

We present a new way of using agent-based modelling to account for the magnitude and diversity of everyday social interactions in the city, and to evaluate their impact on the formation and expression of inequalities in health behaviours. We first review how individual behaviours may be affected by residential-based and activity-based contexts (Sect. 1.1) before presenting social determinants of health behaviours, and more specifically in dietary behaviours (Sect. 1.2), the everyday segregation in cities, and especially in Paris (Sect. 1.3) and previous attempts at modelling health dynamics within a complexity framework (Sect. 1.4). A summary of the main objectives of the paper concludes this introduction (Sect. 1.5).

### Residential-based and activity-based contexts

Where one lives, works and travels plays an active role in the way one thinks and acts. Therefore, the influence of places on individual behaviours has been studied and coined as neighbourhood effects.

#### Neighbourhood effects

describe to the fact that *“neighborhoods are not merely settings in which individuals act out the dramas produced by autonomous and preset scripts, or empty vessels determined by ‘bigger’ external forces, but are important determinants of the quantity and quality of human behavior in their own right.”* [61, p.22]. Neighbourhoods affect people through their physical attributes, their reputation, cultural symbols, and/or through their social composition. The effect on individuals is shared behaviours or outcomes among neighbours. Neighbourhood effects usually apply to socioeconomic outcomes, health, or education. They are hard to identify empirically because individuals adapt to each other, sort themselves between places and because they only live once, which makes alternative trajectories impossible to observe. However, longitudinal research (e.g. van Ham and Tammaru [76]) and policy experiments (cf. *Moving To Opportunities* and its analysis by Chetty et al. [18] or Sampson [61]) have shown some limited but positive evidence of neighbourhood effects. For example, the achievements of similar pupils (same occupation of the parents for example) vary depending on the social composition of their school and residential neighbourhood [2]. Galster [30] summarised neighbourhood effects into fifteen mechanisms, from social interactions (contagion, socialisation, competition) to environmental and geographical processes (toxic exposure, physical constraints, spatial mismatch) and institutions (stigmatisation, market actors). Not all of the above mechanisms are always relevant and they may play out at different scales [56]. In this study, we put the emphasis on the social interactions mechanism which may occur daily in local neighbourhoods and test its role in shifting opinions and eventually impact behaviours.

#### Social segregation and neighbourhood effects around the clock

Neighbourhood effects and local residential environments do exhaust the question of social differentiation in cities. Following calls to avoid the ‘local trap’^1^ [21] and acknowledging differences in the attributes of residential and non-residential neighbourhoods [64], studies of people’s activity spaces and multiple exposures have emerged [40], notably in the health literature, using travel diaries, go-along methods, GPS traces, mobile phone data and so on [55]. This approach is inspired by time-geography [33]. However, there is often a strong discrepancy in this literature between the high degree of accuracy in daily trajectories of people and the low degree of accuracy in daily trajectories of places, as though place attributes suffered from a kind of ‘jetlag’ or ‘clocklag’ [73]. Rather than freezing neighborhood attributes from *“a notional time when all members of the population are at their residential address”* [47, p.755], as is generally done using census-based information to define a neighborhood’s social profile, it is useful to remove ‘clock blinders’ and explicitly consider the ‘daycourse of place’ [73], i.e., the daytime variations in neighbourhood’s social profiles. It may help to explore the everyday life spaces in which individuals develop their sociospatial networks [63] and in which they are susceptible to adapt their health behaviours according to that of the individuals with whom they interact.

### Inequalities in health behaviours: the case of dietary behaviours

One strand of the public health literature assesses whether health policies are effective in increasing healthy behaviours in the population as a whole, while reducing inequalities between social groups (i.e. avoiding the concentration of benefits among the better-offs). Health interventions that are evaluated (and compared with systematic reviews) for their effectiveness in responding to this equity challenge include policies aimed at reducing tobacco consumption [37], increasing physical activity [34] and promoting healthy diets [49].

Here, we choose to focus on inequalities in dietary behaviours and more specifically fruit and vegetable consumption. Increasing fruit and vegetable intake in the general population has become a public health priority in many countries, with government agencies around the world recommending a daily intake of at least five servings of fruit and vegetables (i.e. 400 g/day). In France, several prophylactic campaigns have been launched as part of National Nutrition and Health Program (PNNS). The famous “Eat five fruits and vegetables a day” slogan appeared in France in 2007 with extensive media coverage, in line with several other national campaigns introduced in North America, Europe and Australia, following the recommendations of the World Health Organization’s Global Strategy on Diet, Physical Activity and Health published in 2004. This campaign was not aimed at any particular audience (vulnerable or not), but was broadcast simultaneously on multiple mainstream media.

In France, it has been found that meeting the 5-a-day recommendation was more likely among the female population, for people aged 50+ and individuals with higher education levels [25, 32]. A cross-sectional study in 21 European countries also showed that being female **and** having a high education was associated with increased consumption of fruit and vegetables, showing that gender mediates the relationship between educational level and fruit and vegetable consumption [68].

Inequalities in fruit and vegetable consumption can be seen as the combined result of (i) physical needs, which change over time and across genders and morphologies; (ii) access to fruits and vegetables, which vary according to household resources (income, transportation means etc.) and to service availability in neighborhoods [51]; (iii) tastes and preferences, which differ according to the position of individuals in society [13] and with dietary habits prevailing at the family [38] and neighbourhood levels. Because of the importance of combined identities, as well as personal and household attributes in the realisation of healthy or unhealthy behaviours, we think that intersectionality [19] is a fruitful framework to study social inequality in health. Intersectionality reflects the complex nature of interacting identities which cannot be analysed in isolation, and their consequence on individual and social outcomes through cumulative discrimination and/or privilege. Bauer et al. [11] show that 40% of research using the intersectionality framework in the last 30 years has indeed focused on a health-related outcome.

While accounting for age-based and gender-based inequalities in fruit and vegetable consumption, the present research aims to explore the global evolution in fruit and vegetable consumption as a function of intersecting age, gender and education-based inequalities. We assume that inequalities are maintained and reinforced through the socially-constrained diffusion of opinions and their influence on behaviours in daily environments.

### Everyday segregation in cities: the case of the Paris region

The literature investigating the spatial distribution of social groups over the 24-hour course of the day is limited. This contrasts with the vast amount of authors who have looked into the measurement, description and evolution of social segregation from a residential perspective [52, 76], and of the Paris region in particular [58, 60, 78]. The results of such studies show that individuals at the top of the social hierarchy tend to be the most segregated group. They choose residential locations with restricted access where they and their neighbours can share similar expectations regarding property prices, public services and amenity levels. In the Paris region, these areas of affluence are located to the West of the city (in the 7th, 8th, 16th and 17th arrondissements of inner Paris and the Hauts-de-Seine district, which includes Versailles, Neuilly and Courbevoie). At the other end of the spectrum, the poorest and most vulnerable individuals in society are also more segregated than average on the housing market, because of the spatial concentration of affordable and social housing. In the Paris region, these areas of poverty are located to the North-East of the city (in the 18th, 19th and 20th arrondissements of inner Paris as well as the Seine-Saint-Denis district, which includes Clichy-sous-Bois, Aubervilliers and La Courneuve).

Only recently, authors have started to explore urban segregation from an activity-based approach [26, 54, 66]. In the Paris Region, Le Roux et al. [42] have computed hour-by-hour maps of area-level social profiles from a large origin-destination survey. These profiles, along with segregation indices such as Duncan index of dissimilarity^2^ are displayed in the open and interactive *Mobiliscope*^3^ platform [74]. They give new light on segregation in Paris region around the clock (see Supplementary material, Sect. 1): segregation was found to increase between nigthtime and daytime for people with a very low level of education, to remain fairly stable for those with an intermediate level of education and to decrease for those with a high level of education (three years or more after obtaining their French high-school diploma - Baccalauréat). Some interesting patterns in everyday segregation also appear for attributes such as gender and age: gender-based segregation, segregation of the youngest (aged 25-34) and segregation of the oldest people (65+) was found to increase during the day compared to nighttime residential measurements.

These empirical findings urge us to consider everyday segregation in cities when modelling social interactions and their impacts on social inequalities of health behaviours. However, methodological limitations to the various sources of data (whether surveys or big data such as mobile phones) make it difficult to combine these different patterns of segregation with an intersectional approach and to compare, for example, the everyday segregation of advantaged middle-aged men vs. poor elderly women. Recent analyses of whether peers with similar sociodemographic characteristics are synchronously located over the 24-hour period [75] could usefully be extended to individual-based models with the aim of exploring social interactions around the clock with an intersectional lens.

### Agent-based modelling to test hypotheses regarding social inequalities in health behaviours

Agent-based models (ABMs) are becoming a widespread methodology of social sciences, health studies being no exception. In their review of the general uses of ABM in public health, Tracy et al. [71] identified mainly epidemiology, disease control and health behaviour (tobacco and drug usage, physical activity, diet) as the three main application domains of the methodology. ABMs in the public health literature make very heterogeneous usages of the ABM meta-formalism [72], they operate at various levels of complexity and their representation of mechanisms varies from the KISS (Keep It Simple, Stupid) approach to the KIDS (Keep It Descriptive, Stupid) approach [8]. Finally, different geographic scales (micro/macro) can be observed [4, 5, 53, 79]. For instance, we could find the diffusion and control of infectious disease [5, 79] implemented at macro scale in a system of cities, at micro scale by simulating realistic mosquito flying at neighbourhood level in cities [46], but also at some macro-micro scale using coupled -ODE and ABM- formalisms [7]. When considering the literature more specifically focused on the effects of socioeconomic status on health outcomes, Speybroeck et al. [67] found that out of 61 studies devoted to this relationship and involving some form of simulation in English language, 4 were expressly about agent-based models. These 4 were all spatial, dynamic, multilevel and included heterogenous agents.

In the health and social behaviour context, Auchincloss et al. [3] tackles residential segregation and its effect on access to fresh foods, but does so using a two-zone distribution of the population (e.g. the rich on one side of the grid and the poor on the other side). Langellier [41] models the persistence of social inequalities in health behaviour (in this case: chewing gum) through the effect of segregation, clustering and social influence. Pham et al. [57] use non-spatial social networks to model the effect of social segregation on the planetary spread of the vegetarian diet. However, ABMs of health behaviour and segregation are usually limited by their lack of realism regarding the representation of geography and daily mobility, the uncertainty of their results (due to the model resolution as well as fitting with empirical data) and their limited validation process. These opportunities and challenges apply to our project so we try to address them in the remainder of the project.

### Filling the gap: using ABM to explore the role of everyday geographies and segregation on long-term dynamics of social and spatial inequalities

In this paper, we use an agent-based model to explore the dynamics of social and spatial inequalities in dietary behaviours. We expect the precision of the temporal (everyday) representation of segregation to play a nontrivial role in the simulation of inequalities in dietary behaviours. We also expect individuals’ susceptibility to opinion contagion to vary according to the combination of their gender, age and socio-economic position. We therefore favour an intersectional and realistic representation of agents by using a synthetic population based on empirical data, not only regarding their residential location and daily mobility, but also their dietary opinions and behaviours.

Our objective is to test whether long-term changes in dietary behaviours of the whole population and their unequal distribution between social groups and within urban areas do actually differ (i) when daytime locations are taken into consideration besides nighttime locations and (ii) when nighttime and daytime locations are subject to social segregation (as in real-life) compared to a theoretical scenario where people are located at random.

## Empirical data and preliminary work

This section describes the generation of a synthetic population representative of the spatio-temporal distribution of population in the Paris region (Sect. 2.1) as well as the French data used to analyse dietary behaviours (Sect. 2.2).

### Synthetic population generation: the H24 library

#### 8+ million agents in 8000+ cells

**Figure 1:**
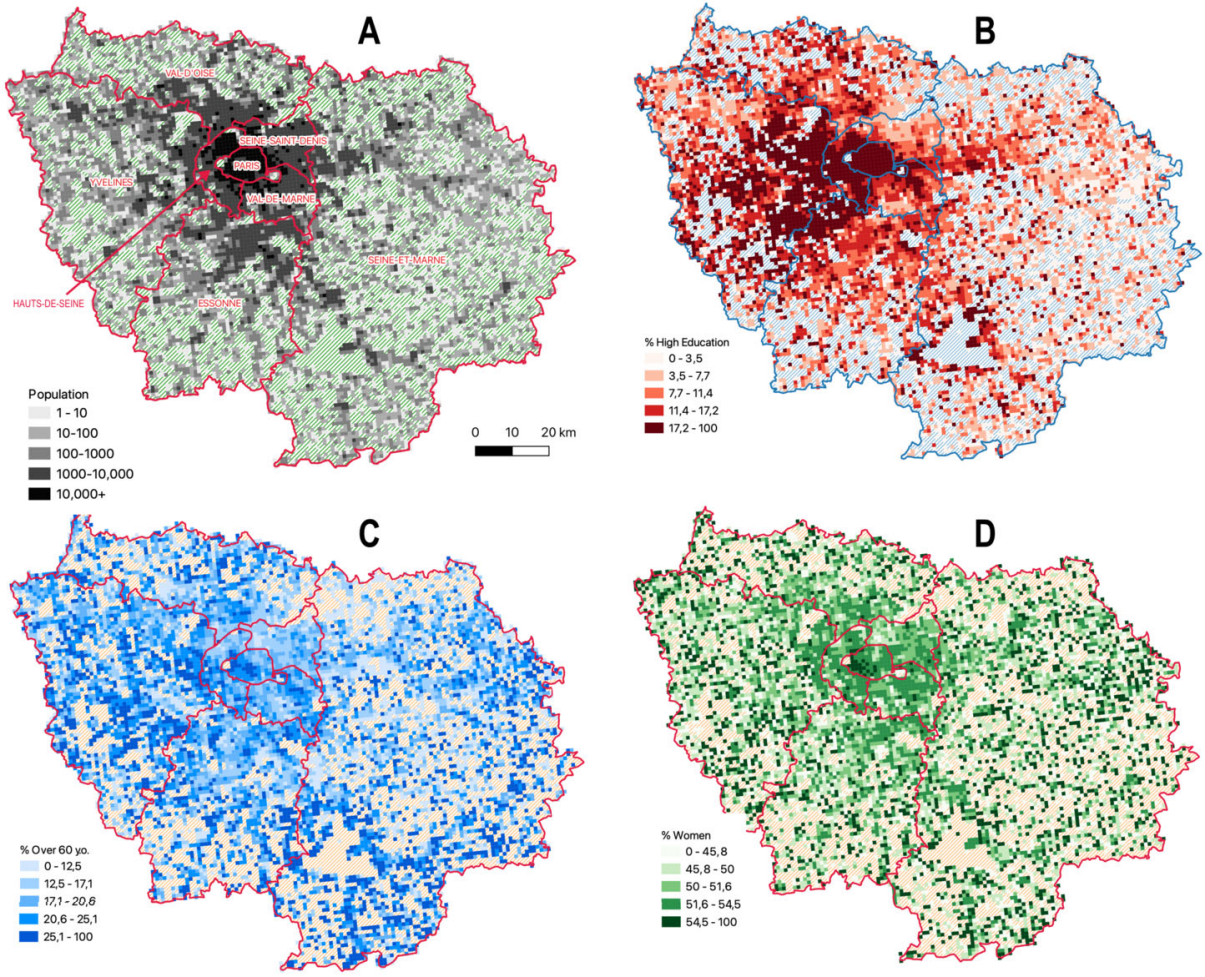
Residential distribution of the synthetic population in the Paris Region according to four sociodemographic attributes

The Paris region (‘Ile-de-France’), with an area of 12,000 km^2^, is divided into a grid composed of 8325 (inhabited) 1 km × 1 km cells (extracted from INSEE 1 km population grid as illustrated in Fig. 1). Around 8.77 million agents (representing the total number of residents aged 15 to 75) are distributed into this spatial grid.

#### Sociodemographic attributes

Since sex, age and education are three individual attributes known to affect dietary behaviours (see Sect. 1.2) as well as everyday mobility and segregation (see Sect. 1.3), every agent in the simulation is described by three attributes: sex (male/female); age (15-29; 30-59; 60-75) and level of education from the last achieved qualification (“low” for those with less than the baccalaureate certificate - Bac sanctioning the end of high-school; “middle” for those from Bac to 2 years of higher education - Bac+2; and “high” for those with 2+ years of higher education). For those still at school, no information was available about their highest achieved qualification. We defined their education level based on their age: “low” for students under 18; “middle” for students between 18 and 24; “high” for students aged 25 and more. More details about final agent distributions in each of the 18 sociodemographic categories are available in the Supplementary material, Sect. 2.

Every agent is located in a cell according to the period of the day: in a ‘night’ cell (00:00-08:00), in a ‘day’ cell (08:00-16:00) and in an ‘evening’ cell (16:00-24:00).

#### ‘Night’ cells

We first assign each agent to a fixed location during the ‘night’ time slice based on the 2012 national census data available in open access. Direct sampling is used to estimate the number of inhabitants of each of the 18 sociodemographic categories in every census block (‘IRIS’). The actual counts are not available in open data at this scale for confidential reasons. Agents in each residential census block are then allocated a 1 km × 1 km cell based on the empirical density distribution of populated areas in the Paris region. More precisely, the agents generated at the census block level are randomly assigned to the grid cells intersecting the census block according to a probability that depends on the intersection area between the grid cell and the census block and on the actual population of the cell. At the end of this process, 8.77 million synthetic agents are located in 8325 cells for the ‘night’ time slice, which corresponds to the residential geography (Fig. 1).

The residential distribution of agents in the Paris region follows several well-known patterns. First, the population density is organised according to a centre-periphery pattern (cf. Fig. 1A), with the highest densities visible within the municipality of Paris and the districts of Seine-Saint-Denis (Northeast), Val-de-Marne (Southeast) and Haut-de-Seine (West). Beyond this dense core, the population is concentrated along river axes such as the Seine, the Marne or the Oise. In terms of education (and income), populations in the West of the Paris region are on average better endowed than populations in the East of the region, with the exception of the area around Fontainebleau (cf. Fig. 1B). Elderly people are present in varying proportions across the region but rather less so in the young district of Seine-Saint-Denis, and more so in the Southwest of Paris or around Fontainebleau (cf. Fig. 1C). Men and women are distributed in comparable proportions across the region, but pockets of predominantly (elderly) female populations can be found in the exclusive southwest arrondissement of Paris 16 and pockets of predominantly (immigrant) male populations can be found in the northern working-class arrondissement of Paris 18 or in Saint-Denis (cf. Fig. 1D). Looking at indices measuring subgroup spatial concentration (Duncan’s dissimilarity index and Moran’s spatial-autocorrelation index), we note that residential segregation is particularly strong for high (and low) educated subgroups compared to age-based or gender-based segregation (see Supplementary material, Sect. 3).

#### ‘Day’ and ‘evening’ cells

For the ‘day’ (08:00-16:00) and ‘evening’ (16:00-24:00) time slices, agent locations are generated using a spatial distribution of daily trips estimated from the origin-destination survey (‘Enquête Globale Transport’ - EGT) carried out in 2010 in the Paris Region. After selecting EGT respondents aged 15+ that were surveyed about their mobility on weekdays (Monday to Friday), the final EGT sample contains over 26,600 respondents, making with a total of 106,100 trips [42]. We describe respondents using the same three attributes as for census data: sex, age and level of education (following the same logic as for census data for those still in school).

We transform the trip dataset into a location dataset (defined by a 100 m × 100 m grid) with temporal information (hh:mn). We transpose each location into the 1 km × 1 km cells covering the Paris region. We consider the home location of respondents as their ‘night’ (00:00-08:00) location. We focus on respondent locations between 08:00 and 24:00 to assign agents to their ‘day’ (08:00-16:00) and ‘evening’ (16:00-24:00) cells using a three-step process.

- First, for every cell of the Paris region, we compute weighted probabilities for an agent to be there during ‘day’ (08:00-16:00) and ‘evening’ (16:00-24:00) time slices based on the cumulative time that respondents with similar sociodemographic characteristics and similar night locations spent there.
- Second, we interpolate the spatial distribution of these weighted ‘day’ and ‘evening’ probabilities into the nearest-neighbour cells that lack them (but only if these cells are populated). This spatial interpolation step provides weighted probabilities for areas close to those visited by the sample of respondents to the origin-destination survey, thereby filling in the ‘white areas’ on the city map that simply reflect the incomplete spatial coverage of the origin-destination survey.
- Third, we use these interpolated weighted probabilities to assign agents to ‘day’ and ‘evening’ cells according to their sociodemographic attributes and to their nighttime location. For agents whose sociodemographic profile and nighttime location refer to missing probabilities (because no probability could be calculated from the survey or spatially interpolated), we use probabilities built for the group of equivalent nighttime location, age, education level, but of the opposite sex. If this information is missing as well, we duplicate the night ‘cell’ into other time slices, making the agents immobile (it concerns around *i.e.* 0.046 % agents in the simulation).

Following this three-step process, 78% of the 8.77 million agents are located in a ‘day’ cell which differs from the ‘night’ cell and around 54% are located in an ‘evening’ cell which differs from the ‘night’ cell (see Supplementary material, Sect. 2). Changes in the locations of agents throughout the day mirror some social patterns of everyday mobility documented in the literature. Among agents who leave their night cell during the day (i.e. those with a ‘day’ cell which differs from the ‘night’ cell), there are relatively more men than women and more highly educated agents than agents with a lower level of education (with the exception of the 60–75 age group). Among agents who leave their night cell in the evening (i.e. when the ‘evening’ cell differs from the ‘night’ cell), there are relatively fewer middle-aged agents than younger or older agents (this is particularly true for women, probably due to the gendered distribution of childcare at home). The everyday geography of our synthetic population also reproduces the temporal variations in segregation observed empirically in the Paris region from the *Mobiliscope* platform [74] based on origin-destination survey data (see Supplementary material, Sect. 1). The values of the Duncan dissimilarity index calculated from the agent locations (see Supplementary material, Sect. 3) indicate, as expected, that segregation based on gender and age is systematically stronger during the day (and also in the evening) than at night. Other examples of correspondence with the empirical patterns include the segregation of older agents, which exceeds that of other younger groups by far during the day, and the lower level of segregation of middle-educated agents compared to those with a higher or lower levels of education, regardless of the time of day.

The H24 library to create such a synthetic population is freely available online.^4^ We also provide a documentation with examples on a companion website.^5^

### Dietary habits

#### Data

The Baromètre Santé Nutrition (Health and Nutrition Barometer) is a recurrent nationally representative telephone survey designed to assess the evolution in behaviour, knowledge and attitudes towards food in the French population (aged 5 to 75). For this study, we use two Health and Nutrition Barometer surveys carried out in 2002 and 2008 (Santé publique France) and we select the respondents aged 15 to 75. We exclude respondents living in rural municipalities outside the Paris region and those with missing data regarding their sex, age or highest qualification achieved. Every respondent is described by three attributes: sex (male/female); age (15-29; 30-59; 60-75) and level of education from the last achieved qualification (“low” – less than bac.; “middle” - bac. to bac. +2; “high” > bac.+2). For respondents still at school, their level of education was defined directly from their last achieved qualification, except for minor students who had not completed high school yet (they were defined as having a middle level of education) and for students below the age of 25 who had graduated from high school (they were defined as highly educated). This process was designed to reduce misclassification that may arise for young adults, i. e. the attribution of a lower educational attainment than that actually achieved later on [29]. Our sample contains 2096 respondents in 2002 and 3304 in 2008.

#### Fruit and vegetable consumption

For the survey, every respondent had to report their food consumption of the previous day. Summing each type of foods, the survey provides estimated frequencies of daily consumption, including that of fruit and vegetables. Based on the 5-a-day reference corresponding to the public health slogan “Eat Five Fruit and Vegetables a day” widely broadcast in France during the 2007 Programme National Nutrition Santé (PNNS), we compute a binary variable corresponding to two possible outcomes: either the respondent has eaten at least 5 portions of fruit and vegetables the day before or the respondent has not. We observe that the proportion of respondents reporting eating at least 5 portions of fruit and vegetables a day was higher in 2008 than in 2002, and that female, older and more educated respondents were more likely than their counterparts to report eating at least 5 portions of fruit and vegetables a day (Table 1). With a population distribution by age and sex corresponding to the Paris region according to the 2012 census, the global proportion of people exhibiting a healthy behaviour (i.e. eating at least 5 portions of fruit and vegetables a day) was found to increase from 9.6% in 2002 to 12.1% in 2008 (Table 1). The proportion of people with a healthy behaviour in 2002 is used to initialise agent behaviour according to their sociodemographic category. The proportion of people with a healthy behaviour in 2008 is used to calibrate the agent-based model.

**Table 1:** Distribution of sociodemographic groups with key variables from Health and Nutrition Barometers

Sex	Age	Education	Number of respondents		% respondents eating fruit and vegetable 5-a-day		Average respondents’ opinion (2002)		% of respondents with constraints (2002 & 2008)	
			2002	2008	2002	2008	Among healthy	Among unhealthy	Habit	Budget
Male	15-29 yrs.	low	52	148	1.9%	2.7%	0.63	0.54	53%	54%
Male	15-29 yrs.	middle	97	223	3.1%	4.9%	0.51	0.55	58%	56%
Male	15-29 yrs.	high	84	137	3.6%	7.3%	0.80	0.56	65%	61%
Male	30-59 yrs.	low	241	355	5.0%	8.2%	0.64	0.53	64%	57%
Male	30-59 yrs.	middle	122	225	7.4%	11.1%	0.61	0.57	84%	48%
Male	30-59 yrs.	high	118	180	5.9%	16.7%	0.74	0.59	83%	43%
Male	60-75 yrs.	low	95	149	14.7%	17.4%	0.60	0.57	58%	53%
Male	60-75 yrs.	middle	31	46	3.2%	23.9%	0.63	0.57	86%	47%
Male	60-75 yrs.	high	28	34	17.9%	20.6%	0.62	0.52	93%	29%
Female	15-29 yrs.	low	66	110	3.0%	3.6%	0.54	0.56	72%	73%
Female	15-29 yrs.	middle	121	252	5.0%	8.7%	0.70	0.59	76%	63%
Female	15-29 yrs.	high	100	195	7.0%	7.7%	0.66	0.61	81%	70%
Female	30-59 yrs.	low	341	369	13.5%	10.6%	0.68	0.60	77%	69%
Female	30-59 yrs.	middle	178	302	14.6%	11.9%	0.73	0.61	85%	66%
Female	30-59 yrs.	high	124	211	14.5%	17.1%	0.69	0.64	89%	59%
Female	60-75 yrs.	low	235	241	20.0%	22.0%	0.64	0.63	71%	72%
Female	60-75 yrs.	middle	46	87	26.1%	31.0%	0.68	0.64	81%	69%
Female	60-75 yrs.	high	17	40	23.5%	30.0%	0.70	0.62	82%	61%
		Total*	*2096*	*3304*	*9.6%*	*12.1%*	*0.66*	*0.58*	*75%*	*59%*

*: with population distribution by age, sex and education corresponding to Paris region from 2012 census

Source: Baromètres Santé Nutrition, 2002-2008 (Santé Publique France).

#### Social inequalities in fruit and vegetable consumption

To explore the dynamics of social inequalities in dietary behaviours, we have devised a bespoke index called $EducIneqIndex$ ($EII$), a ratio of health outcomes between the most and least educated individuals in each age × sex group (because we know that women and older women in particular tend to have very different behaviours compared to men in general, and younger men in particular, regardless of education level), weighted by their share in the population. $EII$ therefore compares the differential proportion of people with a healthy behaviour in the highly educated group versus the low educated group for each age/sex category, accounting for the population distribution by age and sex:

1 $$ EII_{t} = {\sum _{sex=1}^{2}{\sum _{age=1}^{3}{ ( \frac{\%healthy_{sex,age,edu=3, t}}{\%healthy_{sex,age,edu=1, t}} \times \frac{N_{sex,age}}{N}}})} $$

$EducIneqIndex$ ($EII$) always gives positive values. When there is no systematic difference in health outcomes between the most and least educated individuals, the value of the index is 1, which reflects the absence of inequality. When the most educated individuals are systematically healthier than their counterparts in the least educated group (ceteris paribus with respect to age and sex), then $EII > 1$, which indicates high levels of inequality. In contrast, when the least educated individuals are systematically healthier than their counterparts in the most educated group (ceteris paribus with respect to age and sex), then $EII < 1$. In short, the higher the value of $EII$, the larger social inequalities are (i.e. the probability of eating at least 5 portions of fruit and vegetables per day depends to a large extent on one’s sociodemographic category). Empirically, $EII$ was found to increase from 1.408 in 2002 to 1.895 in 2008. $EII$ in 2008 is used to calibrate the agent-based model.

For comparison purposes, we also computed the corrected concentration index $E_{t}$ proposed by Erreygers [24], which measures rank-ordered inequalities in healthy behaviour - and not only differences in healthy behaviour between the more and less educated groups. However, the rank-ordered social inequality index $E_{t}$ ignores the stratification by age and sex (see Supplementary material, Sect. 4), which is crucial in dietary processes.

#### Opinion regarding fruit and vegetable consumption

In 2002 (i.e. before the slogan “Eat Five Fruits and Vegetables a day” was launched), respondents were asked to answer the following question “In your opinion, how many fruits and vegetables should one eat daily to be healthy?”. Based on this question, we create an opinion index. The index value corresponds to the reported number divided by the recommended number (n = 5) or 1 if the reported number is larger than 5. The scale on which opinion is expressed in the model itself is continuous, from “not convinced at all that eating 5-a-day is important” (0) to “fully convinced” (1). We observe that our opinion index varies positively with the measure of fruit and vegetable consumption observed at the individual level: the mean opinion index is 0.66 for people with a healthy behaviour and 0.58 for people with an unhealthy behaviour (see Table 1). Opinion values from the 2002 survey are used to initialise agents’ opinion levels, as a function of their sociodemographic category and the fact that they exhibit a healthy or unhealthy behaviour.

#### Set of constraints

Psychology and behavioural science have demonstrated that people act according to their beliefs (i.e. opinions), but also according to social norms (prophylactic campaigns, family habits) and their perception of control over their behaviour [1]. We assume that external contraints such as family habits and budget limitations act as a filter between dietary opinions and dietary behaviours: one may be convinced that eating 5 portions of fruit and vegetable a day is healthy, but cannot afford it, or lives with a partner who cooks only carbs and proteins.

In the Health and Nutrition Barometer Survey, respondents were asked to report whether household habits and budget had an impact on meal preparation. Combining data from the two Health and Nutrition Barometer Surveys (2002 and 2008), we consider respondents as having habit constraints or budget constraints when they have answered ‘strongly agree’ or ‘somewhat agree’ to these questions. In addition to variations by gender and age group, we observe that respondents tend to report greater habit constraints and lower budget constraints as their level of education increases (see Table 1). For instance (cf. first line of Table 1), 53% of young males with low education have habit constraints and 54% have budget constraints. By contrast, 93% of senior males with high education have habit constraint and 29% have budget constraints.

## The agent-based model (the ‘5aDay’ model)

We model dietary evolution as a dynamic process of opinion change, which is then translated into behaviour under constraints. In this section, we describe how agents are set up at the beginning of the simulation (Sect. 3.1) as well as how they evolve through dynamic rules of mobility, daily exposure to other agents, opinion change and behaviour expression (Sect. 3.2). We present model parameters (Sect. 3.3) and use calibration techniques to check whether our agent-based model can represent the empirical variation of fruit and vegetable consumption observed over the past decade in the Paris region (Sect. 3.4). The code for this ABM (called ‘5aDay’) is freely available online^6^ and the R notebook to generate figures and tables as well.^7^

### At initialisation

At initialisation, each agent is generated with the following attributes: sex, age, education level, a ‘night’ cell, a ‘day’ cell and an ‘evening’ cell. To explore different segregation scenarios, the initial distribution of groups in their ‘night’, ‘day’ and ‘evening’ cells can be generated at random or using the synthetic population described in Sect. 2.1.

We model probabilities of eating 5 fruits and vegetables a day based on the frequency distribution by sociodemographic attributes in the 2002 Health and Nutrition Barometer (Table 1). Those who match the 5-a-day target are defined as “healthy” and the others as “unhealthy”.

Based on this observed behaviour and the distribution of opinions on fruit and vegetable consumption in the Health and Nutrition Barometer 2002 (Table 1), we initialise agents’ initial opinions as a function of their sociodemographic category and behaviour. The distributions of dietary behaviour and opinion attributed to agents at initialisation across the 18 sociodemographic categories are summarised in Supplementary material, Sect. 5.

Some constraints - which will remain fixed during the simulation process - are also assigned to agents at initialisation. These constraints (budget, habits, both or none) are defined according to sociodemographic attributes, based on the 2002 and 2008 Health and Nutrition Barometers (Table 1).

### Opinion and behaviour updates

**Figure 2:**
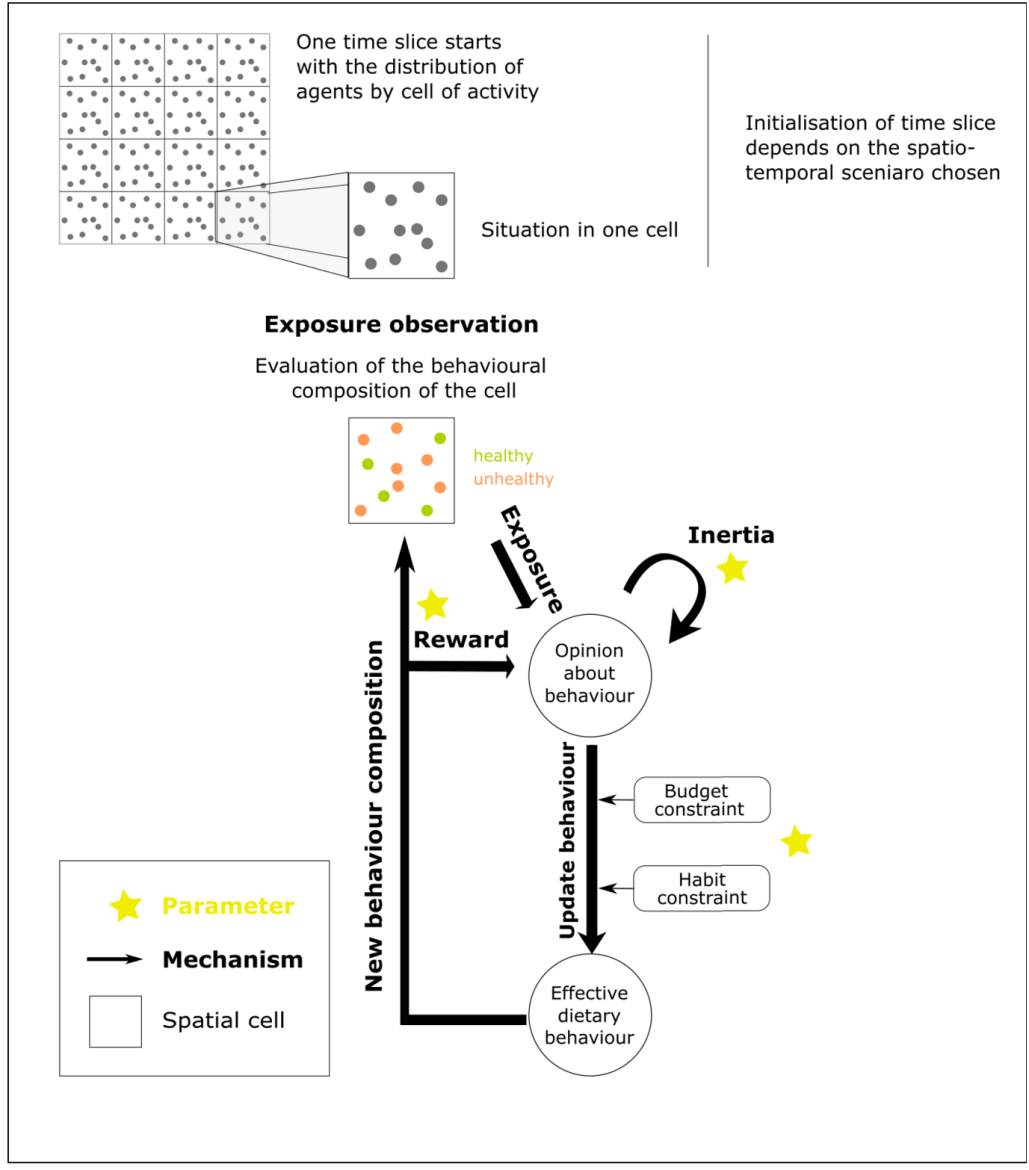
Chain of mechanisms included in the model at each time step

The dynamic part of the model consists of two phases. First, agents are distributed spatially in their cell of current activity (night slice, day slice or evening slice) according to the scenario chosen, the sociodemographic attributes of the agents and the mobility survey described above. Second, when agents are located, a chain of mechanisms starts (as represented in Fig. 2), and ends with an update of both opinions and behaviours. When all agents have been updated, the current time step ends, and a new one can start, possibly requiring a new spatial distribution of agents. The chain involves four mechanisms:

1. **Rewarding behaviour**. Before any movement or interaction, agents reflect on their previous behaviour. If they have been healthy, they feel a positive impact on their physical condition and this reinforces their positive opinion about healthy diets. This “rewarding behaviour” mechanism reflects the positive feedback brought about by the match between one’s behaviour and broader social norms, at a time when national campaigns were launched to promote the health benefits of fruit and vegetable consumption. Since these campaigns did not target any specific audience (vulnerable or not), this reward is modelled at the individual level, regardless of sociodemographic characteristics.
2. **Exposure observation**. Agents scan the distribution of other agents’ behaviours in the cell of their current location and record the information (the % of healthy agents in the cell).
3. **Opinion update**. Each agent updates their opinion on the importance of eating 5-a-day based on their rewarded opinion, some inertia and the current split of behaviours in their current cell.
4. **Behaviour update**. Finally, agents evaluate the opportunity of changing behaviour depending on their updated opinion and the set of constraints which characterise them.

In the subsequent sections, we detail the formalism chosen for each mechanism.

#### Exposure observation

Agents *i* record the behaviour distribution of their current cell *c* and record an exposure value made of the proportion of healthy neighbours (hn) divided by the total number of neighbours (n):

2 $$ exposure\_cell_{i,c} = \frac{hn_{c}}{n_{c}} $$

#### Opinion update, including potential reward

If agent *i* was “healthy” during the previous step, her positive opinion about healthy diets is reinforced by a positive coefficient $healthyDietReward$ (HDR). Agent *i* updates her opinion based on her exposure to healthy individuals in the current cell and reward, if any. This update is subject to inertia (expressed by an $inertiaCoefficient$ (IC)), which prevents agents from changing their opinion radically from step to step.

3 $$\begin{aligned} newOpinion_{i,t} &= \textstyle\begin{cases} IC \times opinion_{i,t-1} + \\ (1 - IC) \times exposure\_cell_{i,c,t}, & \text{if $i$ is unhealthy at $t$} \\ IC \times min(1, (1 + HDR) * opinion_{i,t-1})) + \\ (1 - IC) \times exposure\_cell_{i,c,t}, & \text{if $i$ is healthy at $t$} \end{cases}\displaystyle \end{aligned}$$

This updated value of opinion does not translate directly into a behaviour: norms and constraints can prevent one’s actual diet from reflecting their ideas about diet.

#### Behaviour update

Changes in dietary behaviour occur according to the new opinion agents have about a healthy diet and two types of constraints:

- **Budget** reduces the probability to switch to a healthy behaviour and increases the probability to switch to unhealthy (cheaper) diets.
- **Habits** tend to reduce the probability of switching in both scenarios. For example, an individual might be convinced that a diet different from theirs is better, but their meal schedule is dictated by family preferences or loyalty to certain products.

**Figure 3:**
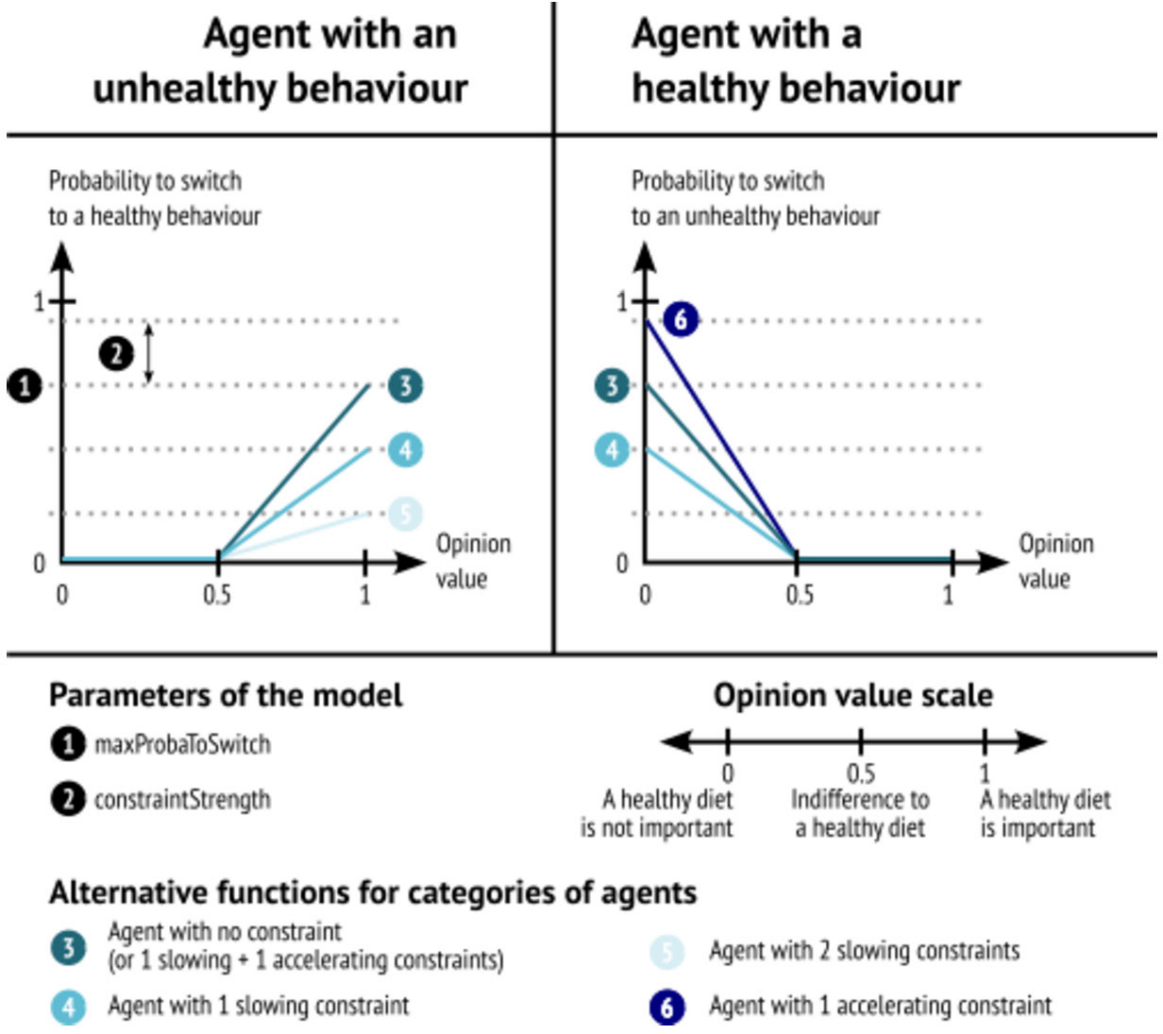
Description of the behaviour switch mechanism in the cases of a healthy agent and in the case of an unhealthy agent

We give the same strength to each constraint and add their effect as in Fig. 3. In the case of an unhealthy agent (left panel), habit and budget constraints both “slow down” the switching process, so we obtain a sum of slowing constraints $n_{u}= \{0,1,2\}$. If the unhealthy agent has an opinion value under 0.5, his behaviour is in line with his current opinion: his probability of changing it is nil. If his opinion value is above 0.5, the probability of switching to a healthy diet depends on the maximum probability to switch ($maxProbaToSwitch$), which is a parameter of the model defined in the interval [0,$1-n_{u}* constraintStrength$]. This maximum probability is reduced by $n_{u}$ times $constraintStrength$, which is a parameter of the model defined in the interval [0,$\frac{maxProbaToSwitch}{n}$].

In the case of a healthy agent (right panel), the habit constraint can “slow down” the switching process and the budget constraint can “accelerate” the switching process, so we obtain a sum of slowing constraints $n_{h} = \{-1, 0, 1\}$. If the healthy agent has an opinion value above 0.5, her behaviour is in line with her current opinion: her probability of switching is nil. If her opinion value is under 0.5, the maximum probability to switch (MPS) can be reduced by $constraintStrength$ (CS) if the agent is subject to habit constraint, or it can be increased by $n_{h}$ times $constraintStrength$ if she is subject to budget constraint (although this constraint is asymmetrical as it is cheaper to switch to a unhealthy diet but more expensive to switch to a healthy diet).

4 $$ y_{i}= \textstyle\begin{cases} MPS - n_{u,i} * CS , & \text{if the behaviour of $i$ is unhealthy}\ \\ MPS - n_{h,i} * CS, & \text{if the behaviour of $i$ is healthy } \end{cases} $$

The function $f(.)$ which gives the probability of switching behaviour *x*, given the maximum probability to switch *y* and the updated opinion *o*, is therefore given by:

5 $$ x_{i} = f(y_{i},o_{i})= \textstyle\begin{cases} max(0, y_{i} * (2 * o_{i} - 1)) , & \text{if the behaviour of $i$ is unhealthy}\ \\ max(0, y_{i} * (-2 * o_{i} + 1)), & \text{if the behaviour of $i$ is healthy } \end{cases} $$

Given this switching probability, we update agents with a new behaviour and let them move to their new activity location.

### Parameterization

The model has different types of parameters: spatio-temporal parameters (which determine the location of agents at different times of the day), model duration, dietary parameters derived from empirical survey results, and free parameters for which we do not have empirical values and which need to be calibrated.

#### Spatio-temporal parameters

This set of parameters comprises different layers. A first parameter related to nighttime locations determines whether the synthetic population is initialised with random nighttime locations (scenarios 1A and 1B of Table 2) or with nighttime locations which match the residential locations observed in the Paris region (scenarios 2A, 2B and 2C of Table 2). A second parameter determines whether synthetic agents remain in their ‘night’ cell (scenarios 1A and 2A of Table 2), move during the day and evening, at random (Scenarios 1B and 2B of Table 2), or as observed in the origin-destination survey (scenario 2C of Table 2).

**Table 2:** Design of the five spatiotemporal scenarios

	Sc. 1A	Sc. 1B	Sc. 2A	Sc. 2B	Sc. 2c
Residential (‘night’ cell)	Random	Random	Observed	Observed	Observed
Daily moves in ‘day’ & ‘evening’ cells	/	Random	/	Random	Observed

Scenarios in which agents do not move during the day are common in the simulation literature on neighbourhood effects [3, 12, 81], mainly because it is the most straightforward to implement. They correspond to scenarios 1A and 2A in our experiments. Scenarios in which agents move during the day according to empirical patterns (such as scenario 2C) are more demanding in terms of data and skills, but they offer a better representation of the complexity of everyday segregation and social interactions. Finally, scenarios using random allocations (such as scenarios 1B and 2B) provide a good benchmark for assessing the effect of sociospatial stratifications on the modelled dynamics. With our setup, we are able to compare scenarios and provide conclusions on the importance of spatiotemporal dynamics representation on the modelled evolution of social inequalities of health.

Our ‘5aday’ model is calibrated (see Sect. 3.4) using empirical data on residential locations and daily mobility (scenario 2C of Table 2).

#### Modelling time

The model runs for six series of three time steps. The three time steps represent the daily slices of mobility and interaction (day, evening and night). We model them as representative of a year of daily mobility and interaction, and therefore simulate them six times to represent the six years of empirical evolution between 2002 and 2008.

#### Survey-calibrated dietary parameters

The survey-calibrated parameters correspond to observations from the 2002 and 2008 Health and Nutrition Barometers (Table 1), such as:

- Initial probabilities of eating 5-a-day in 2002 for each sociodemographic group.
- Initial values of opinions about a healthy diet, for each sociodemographic group, according to the observed distribution of opinions in 2002 (in quintiles) among respondents with a healthy behaviour or unhealthy behaviour.
- Probabilities of being affected by each of the two constraints, for each sociodemographic group, computed from the 2002 and 2008 Health and Nutrition surveys.

#### Free parameters

Finally, four free parameters (Table 3) need to be calibrated.

**Table 3:** Summary of the four free parameters in the model

Parameter	Mechanism	Range	Effect if min	Effect if max
*healthyDietReward*	Opinion	[0 , 1]	No Reward	Behaviour determines opinion
*opinionInertia*	Opinion	[0 , 1]	Stable opinion	Opinion depends on others
*maxProbaToSwitch*	Behaviour	[0, 1 − *n*∗*constraintStrength*]	Stable behaviour	Switch probability is a linear function of opinion
*constraintStrength*	Behaviour	[0 , $\frac{maxProbaToSwitch}{n}$]	No constraint	Constraints prevent switch

We expect the first parameter ($healthyDietReward$) to be positively correlated with the divergence of behaviours observed within a simulation, as the larger it gets, the more healthy agents will be locked-into their healthy behaviour. We expect the second parameter ($opinionInertia$) to influence the effect of segregation on diet inequality. Indeed, it controls the influence of others on a given agent’s opinion. It also makes the spatial dependency explicit, which should be apparent when comparing the different scenarios. The third parameter ($maxProbaToSwitch$) should affect the speed of convergence of the model, which is not the main element we are interested in with this exploration. Finally, the strength of constraints ($nconstraintStrength$) should highlight the impact of sociodemographic attributes on dietary behaviours and be positively correlated with them. Indeed, it would prevent the expression of the opinion mechanism by locking constrained groups into their initial behaviour or bias them towards an unhealthy diet.

### Model evaluation

Our model is calibrated using the scenario where locations in the ‘night’, ‘day’ and ‘evening’ cells match observed residential and daily patterns (scenario 2C in Table 2). To calibrate our model (and thus define values of the four free parameters), we aim to minimise the distance between simulated data and observed data (from 2008) about the number of healthy agents, and about social inequalities in dietary behaviour. To achieve these two objectives, we compute $\Delta _{Health}$, which is the difference (in absolute value) between the simulated and observed numbers of healthy agents, and $\Delta _{EII}$, which is the difference (in absolute value) between the simulated and observed social inequalities in dietary behaviour, as expressed by $EducIneqIndex$ (EII).

6 $$\begin{aligned}& \Delta _{Health} = \sum _{sex=1}^{2}{\sum _{age=1}^{3}{\sum _{edu=1}^{3}{|Nbhealthy_{sex,age,edu,simulated} - Nbhealthy_{sex,age,edu,observed}| }}} \end{aligned}$$

7 $$\begin{aligned}& \Delta _{EII} = |{EII_{simulated} - EII_{observed}|} \end{aligned}$$

**Figure 4:**
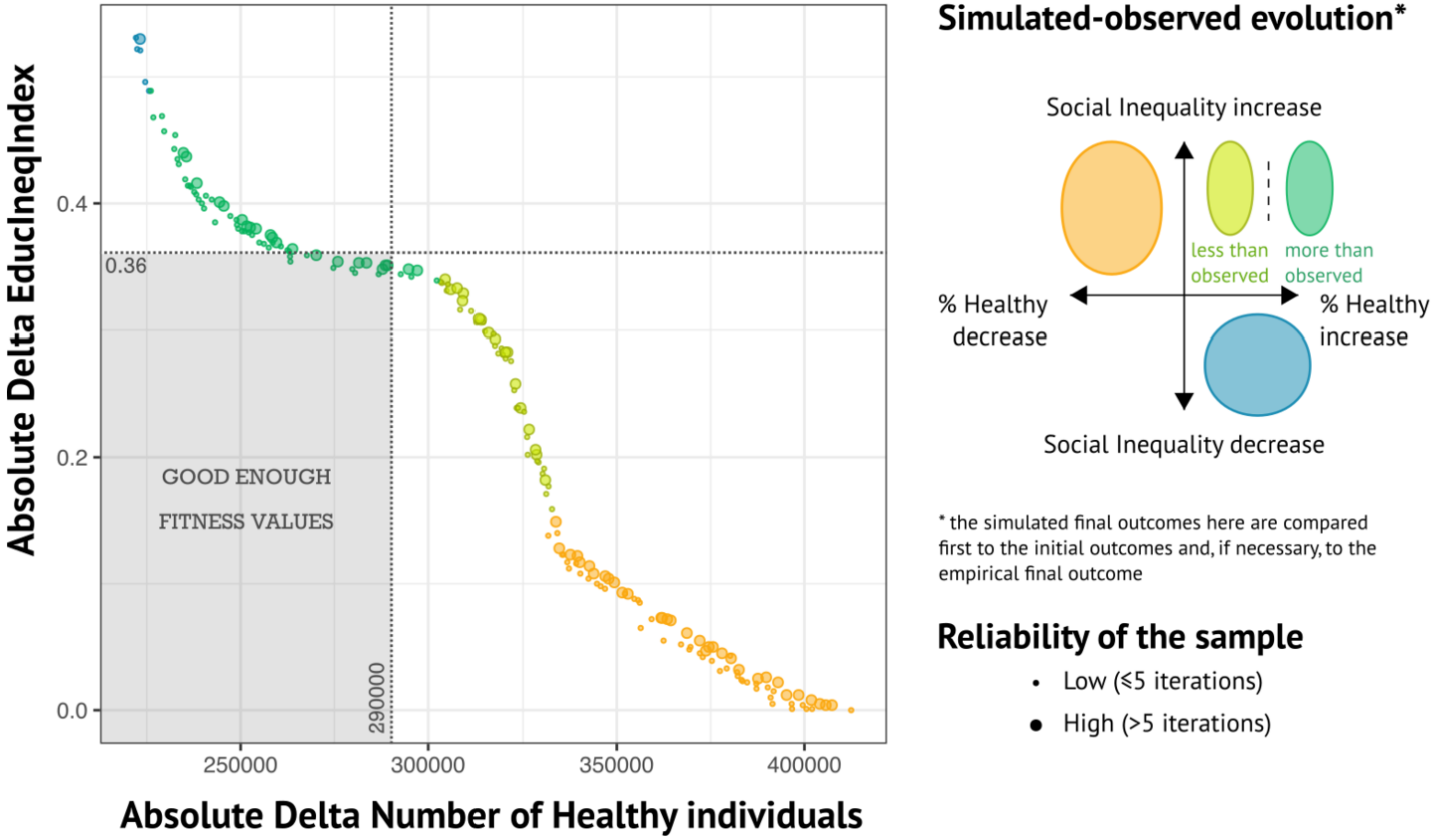
Pareto front of the 197 optimum sets of parameters

In a first step, we use the OpenMOLE framework [59] to perform a calibration process using the NSGA2 optimisation algorithm [22]. The objective of our calibration process is to minimise both $\Delta _{Health}$ and $\Delta _{EII}$. After 376,800 executions of the model, we get 197 optimum sets of parameters with $\Delta _{Health}$ values between 223,000 and 407,000 and $\Delta _{EII}$ values between 0.004 and 0.530, as observed along the Pareto front (Fig. 4).

Four patterns may be distinguished along the Pareto front (see also Supplementary material, Sect. 6 for more details). In terms of robustness, Chérel et al. [15] showed that a model which can lead to a wide array of outcomes is stronger than a model for which all parameters systematically lead to the same results. However, to be in coherence with the dynamic in dietary behaviour observed in the data, we decide to exclude blue sets (in Fig. 4) because they lead to a decrease of social inequality and also orange dots leading to a decrease in the proportion of healthy agents. The remaining green dots (whether a darker or lighter shade) lead to an increase in social inequalities and an increase in the proportion of healthy agents, as observed empirically. Simulated $EII$ is lower than the empirical value of 1.895 in 2008 (but higher than the initial value of 1.408 in 2002) for both dark and light green sets. $\Delta _{EII}$ is greater for the dark green set (from 0.34 to 0.49) than for the light green set (from 0.16 to 0.33). This gives “at best” a simulated EII of 1.55 for the dark green set and of 1.73 for the light green set. $\Delta _{Health}$ is smaller for the dark green dots than for the light green dots. Out of the 8.77 million agents considered, $\Delta _{Health}$ ranges for the dark green dots from 226,000 to 302,000 misclassified agents (2.6% to 3.4%) in terms of their health behaviour, while it ranges for the light green dots from 303,000 to 332,000 ‘misclassified’ agents (3.5% to 3.8%). What distinguishes dark green and light green dots is that the simulated proportion of healthy agents is either higher (darker shade) or lower (lighter shade) than the observed value of 12.1% in 2008. Another notable difference that stands out is the variation in the values of the free parameters. Specifically, the value related to the *healthyDietReward* is close to zero for the light green dots, whereas it is around 0.2 for the dark green dots (see Supplementary material, Sect. 6). Taking all this into account, we choose to retain dark green dots where the ‘rewarding behaviour’ mechanism matters and where the simulated trends in the proportions of healthy agents and $EII$ are acceptable compared to empirical trends. According to this selection, we define *good-enough fitness values* as follows: $\Delta _{Health}$ should be lower than 290,000 and $\Delta _{EII}$ lower than 0.36 (see horizontal and vertical dashed lines in the Fig. 4). We are thus looking for parameter sets which simulate values of $EII$ greater than 1.535 and a misclassify less than 290,000 agents out of the 8.77 million considered (3.3%).

In a second step, we compute the full subset of the parameter space meeting these *good-enough fitness values*. We use here an optimisation method with adaptive rejection zones called OSE (Origin Space Exploration). This method is a modified version of NSGA2, which computes a maximally diverse set of parameter values that all lead to “good” simulations, in our case simulation with values of % health and EII located in the grey area of the Fig. 4. More details about OSE methods can be found in the Supplementary material, Sect. 7. After 517,000 model replications, OSE provides five parameter sets which meet *good-enough fitness values* (Table 4). Among these five maximally diverse parameter sets, we actually observe very small variations compared to their initial definition extent (Table 3): they differ by less than 0.03 for *opinionInertia*, *maxProbaToSwitch* and *constraintStrength*, and by less than 0.09 for *healthyDietReward*. We finally select the parameter set with an intermediate value of *healthyDietReward* and use it to initialise the model presented in the results section.

**Table 4:** The five maximally diverse parameter sets issued from OSE, and the selected one*

	set #1	set #2	set #3	set #4*	set #5
*healthyDietReward*	0.116	0.118	0.129	**0.153**	0.203
*opinionInertia*	0.813	0.799	0.798	**0.820**	0.821
*maxProbaToSwitch*	0.904	0.905	0.898	**0.890**	0.885
*constraintStrength*	0.126	0.126	0.127	**0.129**	0.128

It is important to note that the two exploration processes (NSGA2 and OSE) require around 900,000 executions of our model, each execution lasting about 20 minutes on a single standard execution core. This constitutes a total workload of 35 $years.CPU^{-1}$. To complete this huge amount of computation, we have set up OpenMOLE to delegate it to the 2000 cores of the virtual organisation *vo.complex-systems.eu* of the EGI European grid. The results were available in approximately 6 days for each optimisation method.

## Results

After 10,000 replications of our ‘5aDay’ agent-based model, the proportion of agents with a healthy diet increases from 9.57% (at initialisation) to a median of 13.41% at the end of the simulation. Value of $EII$ increases from 1.405 (at initialisation) to a median of 1.536 as at the end of the simulation. As a reminder, the empirical proportion of respondents with a healthy diet increases from 9.6% in 2002 to 12.1% in 2008 and $EII$ moves from 1.408 in 2002 to 1.895 in 2008. The divergence in target attainment (a simulated healthy diet rate that is higher than the empirical value and a simulated $EII$ that is lower than the empirical value) was expected given the Pareto front (Sect. 3.4). These simulated values correspond to the scenario built to match the observed residential and daily patterns (scenario 2C). For each of the other four scenarios, 10,000 replications of our ‘5aDay’ agent-based model are run to allow us to compare the simulated values of social inequalities (Sect. 4.1) and proportion of healthy agents (Sect. 4.2).

### Social inequalities

#### Comparing the five scenarios

**Figure 5:**
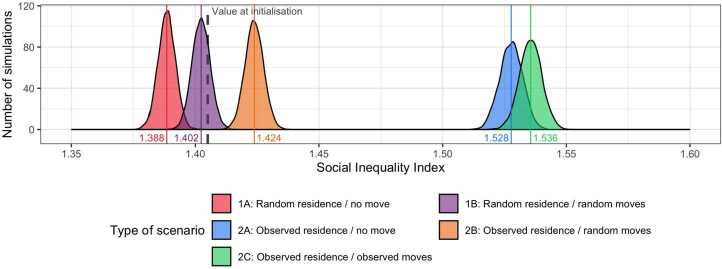
Density distribution of the Social inequality index ($EII$) simulated in the five scenarios (10,000 replications per scenario)

Four main findings can be underlined when comparing simulated values of $EducIneqIndex$ ($EII$) between the five scenarios (see Fig. 5).

First, simulated inequalities in dietary behaviour are smaller than the initial level of inequality when the nighttime locations are chosen at random (sc. 1A and most instances of 1B), and larger when nighttime locations correspond to empirical data (sc. 2A, 2B and 2C). This first result indicates that the absence of segregation at the place of residence leads to a decrease of social inequalities in dietary behaviour over time (based on the spatial and social interactions assumptions specified in our agent-based model).

Second, two distinct sets of social inequality values emerge from the figure: scenarios 1A, 1B and 2B on the left, with significantly lower simulated social inequality values than those simulated for scenarios 2A and 2C on the right. What the three scenarios on the left have in common is that they include random locations (for residence and/or daily moves), leading to a greater mixing of people’s opinions and behaviours at night and/or during the day. On the contrary, scenarios on the right follow empirical patterns only, with or without daily mobility, and result in the persistence of social inequalities.

Third, among the three scenarios in which residential locations correspond to empirical patterns (sc. 2x), social inequalities in dietary behavior are significantly lower when daily moves are randomly defined (sc. 2B) rather when daily moves are ignored (sc. 2A) or defined from empirical data (sc. 2C). This third result leads us to conclude that mixing social groups during the day has the power to mitigate the increase of social inequalities in dietary behaviour induced by residential segregation.

Fourth, simulated values of social inequalities ($EII$) in dietary behaviours are slightly higher when effective daily moves are considered (sc. 2C), compared to when agents remain located at their residential locations during the day (sc. 2A). Daytime segregation leads to an small increase of inequalities in dietary behaviours between the most and least educated groups. This last finding underlines that daytime segregation as it exists in Paris slightly reinforces the unequal distribution of health behaviour between the most and least educated groups already induced by the effect of residential segregation alone.

#### Sensitivity and robustness

**Figure 6:**
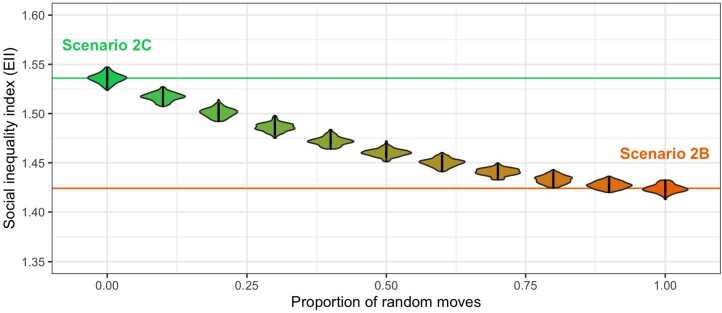
Sensitivity of simulated values of Social inequality index ($EII$) to random moves (vs. observed)

To explore how sensitive simulated values of social inequality are to random *vs.* observed daily moves, we launched variants of scenario 2C, gradually increasing the proportion of random daily moves from 0% (corresponding strictly to scenario 2C) to 100% (corresponding to scenario 2B, see Fig. 6). The relationship between social inequality and the ratio of random moves follows a convex curve. This suggests that social inequality in dietary behaviours decreases with small proportions of stochasticity in daily mobility — that is to say, as soon as spatial segregation decreases slightly. Consequently, only a small proportion of random moves would be required to mitigate the impact of segregation on social inequality in dietary behaviours.

To ensure robustness of our analyses, we also compare our findings using $EducIneqIndex$ ($EII$) with those using an alternative measure of social inequality: the rank-ordered inequality index $E_{t}$ (Supplementary material, Sect. 4). The same trends are generally observed with the rank-ordered index $E_{t}$, except that simulated $E_{t}$ values increase in all scenarios, and are smaller when effective daily moves are considered (sc. 2C) rather than when agents remain located at their residential locations during the day (sc. 2A). Contrasting patterns observed with $EII$ or $E_{t}$ values may be related to the importance given to the middle-educated group in the computation of social inequalities. Whereas $EducIneqIndex$ ($EII$) compares only the difference in dietary behaviour between the least and most educated groups, the rank-ordered inequality index ($E_{t}$) also includes the middle-educated group. However, the rank-ordered social inequality index $E_{t}$ ignores the stratification by age and sex, while $EducIneqIndex$ is weighted by the importance of demographic groups (age × sex).

### Proportion of healthy behaviour

#### Comparing the five scenarios

**Figure 7:**
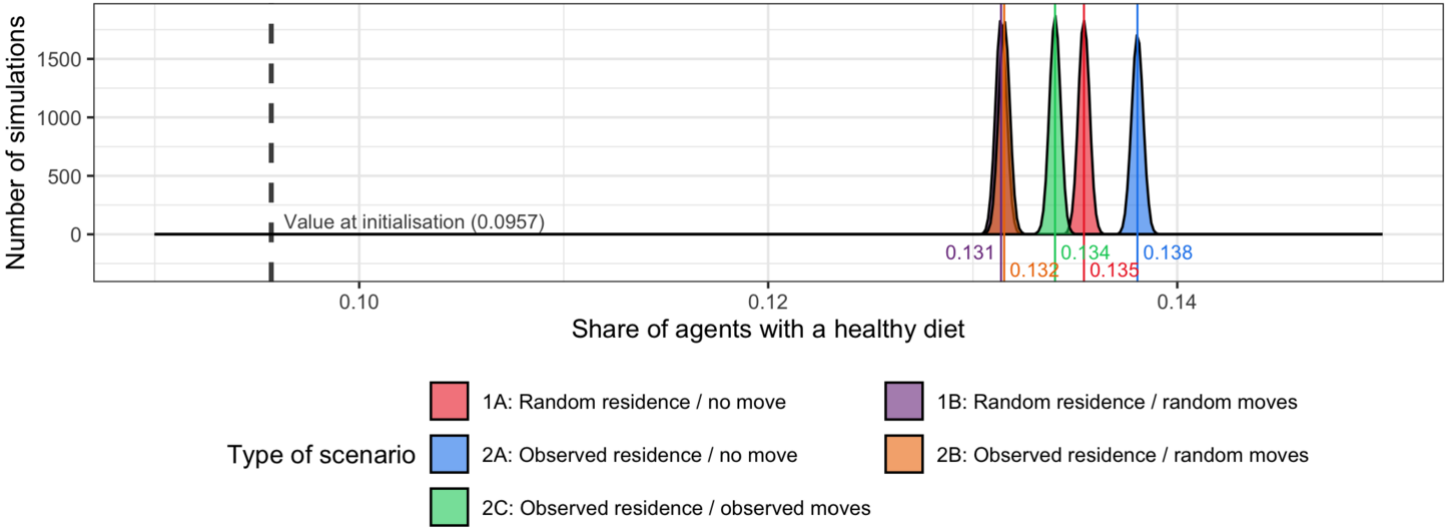
Density distribution of the final proportion of healthy agents simulated in the five scenarios (10,000 replications per scenario)

There are no large variations in the median values of simulated proportions of healthy agents for the five scenarios. They fluctuate around 13,4% with a small difference of 0.67 in percentage point between the lowest and highest median values (Fig. 7). All these simulated proportions are well above the initial proportion of healthy agents of 9.57%. Consequently, changes in the spatiotemporal locations of agents modelled with our five scenarios have a larger impact on the extent of social inequalities in health behaviours than on the overall proportions of healthy agents.

Furthermore, there is no clear relationship between the simulated values of social inequalities and the simulated proportion of health agents for the five scenarios. As an example, we can see that the intermediate values of 13.41% (sc.2C) and 13.56% (sc.1A) correspond respectively to the scenarios where the maximum and minimum values of social inequalities are found (Fig. 7).

The small differences in the proportions of healthy behaviour between the five scenarios may be related to the ‘rewarding behaviour’ mechanism included in the model. This mechanism reinforces positive opinion towards healthy diets for all agents regardless of their social attributes (unlike mechanisms that depend on habit and budget constraints, or locations and their sociodemographic composition). To reinforce this explanation, we can mention the fact that the dots along the Pareto front (corresponding to optimal parameter sets), which are associated with a null ‘rewarding behaviour’ mechanism, led to an increase in healthy behaviours that was less than that observed empirically in 2008 (Sect. 3.4). The ‘rewarding behaviour’ mechanism could therefore be implicated in the overall increase in healthy behaviours over time similarly in all five scenarios. Since we used a fixed level of reward to compare the different scenarios, we therefore control (to some extent) the proportion of healthy agents across scenarios and make more room to variations of social inequality and its association with segregation patterns.

#### Spatial distributions (in ‘night’ cells)

**Figure 8:**
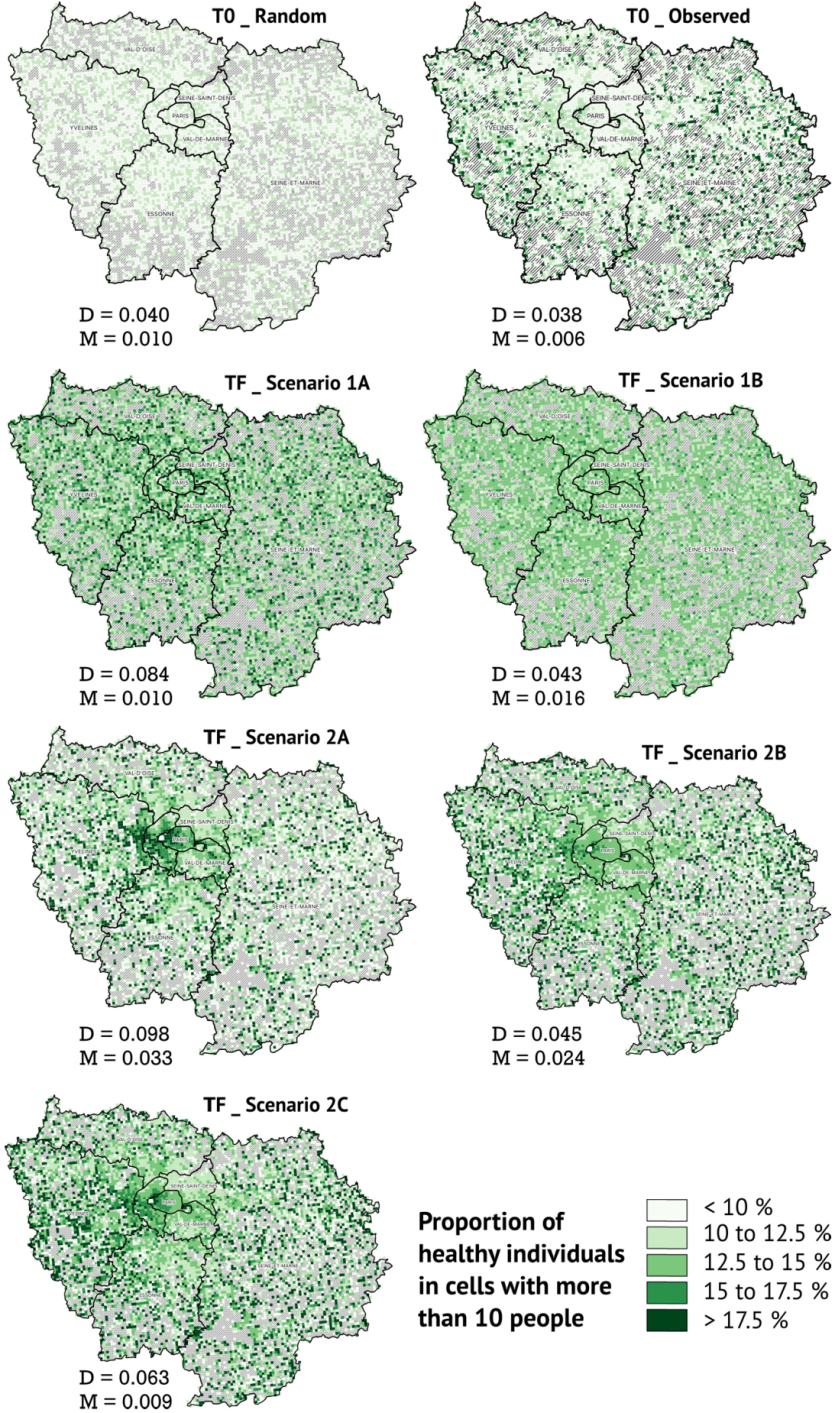
Spatial distribution of agents with a healthy behaviour in their ‘night’ cells: initial (T0) and final (TF) states for the five scenarios, with values of Duncan index of Dissimilarity (D) and Moran’s spatial-autocorrelation index (M) (1 replication of the model)

There is no strong pattern in the spatial distribution of “healthy” agents located at their ‘night’ cells (whether random or observed) at the beginning of the simulation (cf. the first two maps in Fig. 8). At the end of the simulation, scenarios with random ‘night’ locations (i.e. scenarios 1A and 1B) do not exhibit any spatial concentration of healthy agents (cf. Fig. 8): their values of Moran’s spatial autocorrelation of the proportion of healthy agents are close to 0. Actually, in the scenario with no movement during the day (1A), the percentage of healthy individuals has crystallised at random across ‘night’ locations over the course of the simulation, whereas for scenario 1B, random moves during the day resulted in smoothing the percentage of healthy agents across ‘night’ locations at the end of the simulation.

By contrast, we note a strong residential concentration of healthy agents in scenarios where the population is initialised according to the actual patterns of residential segregation in the Paris region, without accounting for actual daily moves (Scenarios 2A and B). These scenarios generate the largest values of Moran’s indices (respectively 0.033 and 0.024) and high proportions of healthy agents in cells of the West of Paris and the Hauts-de-Seine district (where the population is on average better educated, older and more frequently female, i.e. categories with the highest propensity of adopting a healthy diet), whereas the younger, less educated and more male dominated districts of Seine-Saint-Denis and Val-de-Marne show the lowest concentrations of healthy individuals in the model. The spatial contrast is higher in scenario 2A, where individuals do not move during the day (which means that only residential segregation plays a role), and it is lower in scenario 2B, where individuals move at random during the day (which means that the effect of residential segregation is smoothed out by chance encounters during the day).

In the scenario where people follow observed daily moves (scenario 2C), we found cells with high proportions of healthy agents clustered together in the West of Paris, but also scattered in the periphery of the region. Such spatially scattered distribution - corresponding to a lower value of Moran’s I of 0.009 - suggests that daily mobility as it is observed empirically in the Paris region affects individuals in such a way that one’s behaviour becomes less similar to residential neighbours (who might move to different areas and interact with different people during the day).

Combining social, spatial and temporal distributions of dietary behaviours, we can conclude that the observed daily mobility in Paris leads to: 1/ a greater variety of dietary profiles between neighboring areas at nighttime (especially in the periphery of the region); 2/ a more unequal distribution of dietary behaviours between the most and least educated groups, compared to what residential segregation alone can produce.

## Discussion and conclusion

Despite promising findings and insights about the impact of spatio-temporal urban segregation on social nutrition dynamics, our analysis could be improved on a number of dimensions. (Sect. 5.1). However, we think this study is a proof of concept to account for the variation of population in different parts of the cities throughout the day, at scale, which offers opportunities to lead a wide range of analyses (Sect. 5.2). Finally, we summarise the main findings of the study in relation to public policies (Sect. 5.3).

### Limits

#### The characteristics of agents do not evolve

We assumed, in the same way as Zhang et al. [81], that population characteristics (age group, gender, and education level) remain constant throughout the simulation and that agents did not change their residence or workplace. We estimate this caveat to be significant, as at the end of the 6-year simulation, about 17% of the initial agents should be in an upper age category^8^ (which have different probabilities of interacting and a different set of constraints). New agents should appear by crossing the lower age threshold of 15 while some existing agents would die.

#### Aggregated distributions of population subgroups were used to build the synthetic population

The synthetic population generated is the result of a disaggregation of the joint distributions of age, sex and education levels; the agents created should be considered as representatives of their category rather than individuals *per se*. Therefore, the choice of agent-based modelling as a methodological approach, giving precedence to heterogeneous individuals and their interactions, is not fully exploited. However, it gives us the opportunity to estimate the influence of space-time co-locations of heterogeneous agents at scale, i.e. at the scale of the actual Parisian population rather than a representative sample. In the future or other national contexts, microdata on behaviour and sociodemographics could solve this caveat and fully operationalise the intersectional agenda.

#### Some dimensions of interpersonal influence on diet dynamics are left aside

We have not considered household units. However, the household structure, culture and family status influence the food shopping and meal planning of their different members. For example, in France, families with children tend to exhibit a consumption of organic foods which is similar to single females and very different to single males [9], whereas migrant females are more prone than French-born females to have two meals per day instead of three [44]. The synthetic generation of populations into households, although a time-consuming process, is possible [10, 43], and could be implemented when refining the mechanisms of the model to include household dynamics. Besides the household context, we could also have included information on the social context of meals from the two Health and Nutrition Barometer Surveys (2002 and 2008). Actually, 6% of respondents said they ate with someone other than a member of the household during breakfast, 30% during lunch and 13% during dinner. In the future, we could use this information to assign agents different probabilities of interacting about food (and therefore of changing their minds) during the three different time slots considered in the model (night, day and evening). More broadly, we have not fully considered components of “food day” patterns questioned in the sociology of food to compare meals or food intakes according to *“the spatiality of their preparation and consumption”* [at home / out of home] *“and their sociality”* [alone / in company] *“as well as their temporal distribution across the time of the day”* [50, p. 5].

#### Other determinants of food consumption (and segregation) are ignored

Some other individual and place-based determinants of dietary behaviour have been ignored in our particular model. For example, the distribution of shops and equipment could influence purchases and perception of agents [3], whereas some health conditions induce dietary changes in individuals [20]. Income is also not taken into account in addition to education, even though the two may have additive or synergistic effects on dietary behaviours [65]. Income and wealth are important factors in shaping social segregation. Unfortunately, income and wealth data are not available at an individual level in the survey data that we use to generate the synthetic population. Fortunately, however, we know that income covaries with gender and educational level, and wealth covaries with age and educational level. We are therefore confident that education, combined with gender and age, provides sufficient explanatory power without the need to add another factor relative to socioeconomic position. Furthermore, the combination of education, gender and age enables us to deal with a manageable number of social groups (18).

#### Opinion dynamics are basic

The model of opinion change could be refined to particularise the conditions under which agents are receptive to the influence of others. Hegselmann et al. [36], for example, shows the influence of a parameter of bounded confidence (i.e. who an agent trusts to influence their opinion, based on the distance between their initial opinions) and its symmetric/asymmetric interval on the number of consensual opinions. Alternative ways of *‘How, when and where [...] spatial segregation induce opinion polarization’* could also be explored [27].

#### Temporality

Each simulation runs for six theoretical “days” (each with three time slices - ‘night’; ‘day’; ‘evening’ for every day) representing a six-year period. Our model was designed to explore how social inequalities in dietary behaviour simulated at the end of the six-year period could vary according to the spatial segregation of people at night and during the day. It is therefore important to resist the temptation to produce and interpret results beyond the six-year period for which our model was constructed and calibrated. It is difficult to know how findings would differ if the simulation were run with a longer duration or with a different operationalisation of temporality. When exploring the evolution of model outcomes over the six theoretical “days” (see Supplementary material, Sect. 8), we noted an increase in favourable opinion towards healthy eating and an increase in the proportion of healthy eaters, which was slightly faster at the start and at the end of the simulation. These trends suggest that it would be interesting to add a third time point for a better fit of the model, even if this would make the calibration process more complex and time-consuming.

#### Dated data

The last mobility survey in the Paris region with a large number of surveyed residents and a good spatial coverage dates back to 2010. A new mobility survey, scheduled to run for 5 years, was launched in 2018, but the COVID-19 pandemic brought it to a halt. Similarly, our data on dietary opinions and behaviours date back to the previous decade, but have the advantage of matching the survey data and corresponding to a flagship public health campaign. All in all, our model is not time-dependent and could be initialised with more recent data in the future to analyse the impact of more recent policies.

### Opportunities

#### H24. A reusable library for synthetic population generation

H24 is a fully independent and freely available library we developed to generate synthetic populations from census data and origin-destination surveys. A synthetic population throughout the day can be useful when daytime variations in the population present within metropolitan areas are needed, typically to assess population exposure to external physical nuisance or to optimise a given service offer based on current population demand. The H24 library can also be used when the diversity of co-locations of population subgroups (and the resulting social interactions) throughout the day merit attention, e.g. when modelling diffusion or contagion mechanisms (e.g. epidemic spreading or voting models). Even though some literature has recently emerged to model population distribution for nighttime and daytime periods [e.g., 28], it is often restrictive to some population segments (e.g. active population when considering commuting statistics) or socially blind (e.g. without any information about individual attributes, notably when using mobile phone records).

#### A new way to explore vulnerabilities combining traditional survey-based data and agent-based modelling

Information about vulnerable populations are often absent in commonly used big-data sources. Combining conventional mobility survey data, health behaviour survey data and agent-based modelling with this level of spatiotemporal granularity is a promising way to study the well-being of vulnerable populations and to quantify the dynamics of systemic inequalities.

#### Data-driven exploratory model

More realistic than toy models, our project allows a high level of customisation to explore hypothetical segregation situations which differ from an empirical one, such as a random distribution or an immobile population. This feature offers the possibility to explore and compare stark contrasts in scenarios which are still realistic with respect to the size and diversity of the population.

#### Neighbourhood effects around the clock

A very original feature of our project is the mixed use of temporal scales. Indeed, we combine the influence of socially differentiated mobility patterns at the daytime scale (where people reside, go to work and relax) on behaviour inequality with an empirical evaluation which spans several years. By analysing the long-term implications of the social segregation prevailing in cities around the clock, the present research contributes to the debate on the mechanisms of the production of social inequalities in health, and notably on the paradox of the persistence of socioeconomic inequalities in health despite highly developed ‘welfare states’ in Western Europe [45].

### Concluding remarks

Our study is driven by two observations related to public policies: 1/ social segregation resulting from daily activities at various locations affects individuals’ social exposure, interactions and behaviours throughout the day, yet is rarely considered by local policies, which most often consider only residential segregation; 2/ national policies are often designed and evaluated according to their effectiveness in promoting healthy behaviours across the entire population, and pay less attention to equity concerns between social groups (i.e. the concentration of benefits among those who are already better-off).

In light of these observations and to retain complexity on the social, spatial and temporal dimensions, we use an agent-based model including millions of artificial agents moving and interacting in a realistically-sized artificial urban space to measure and explore the effects of social segregation around the clock on the extent of social inequalities in health behaviours in the Paris region. More specifically, we examine whether long-term changes in the dietary behaviours among the whole population, as well as the unequal distribution of these behaviours between social groups, differ according to people’s residential location (random vs. census-based allocation, reflecting the empirical levels of residential and daytime segregation, respectively) and their daily mobility patterns (no daily moves, random moves, or survey-based daily moves, reflecting the empirical levels of daytime segregation in Paris).

Four main findings emerge regarding the social distribution of dietary behaviors: 1/ the absence of residential segregation leads to a decrease of social inequalities in dietary behavior over time; 2/ random locations (for residence and/or daily moves) lead to a greater mix of people’s opinions and behaviours, and therefore to lower social inequality; 3/ mixing social groups during the day has the power to mitigate social inequalities in dietary behavior induced by residential segregation, with this mitigation effect appearing as soon as a small proportion of daily moves are random; 4/ daytime mobility and segregation, as observed in Paris, slightly reinforces the unequal distribution of health behaviours between the most and least educated groups induced by residential segregation. All these findings may be of interest to policymakers concerned with the everyday mechanisms linking spatial segregation, daily interactions and social inequalities.

Finally, it is worth noting that these contrasting results regarding the social distribution of dietary behaviours emerged despite a similar overall increase in healthy behaviours. This suggests that our model can reproduce both the diffusion of dietary behaviours across the entire population and the emergence of distinct diffusion patterns within social groups by simply varying where individuals live and how they move around on a daily basis. This is an important consideration for national policies aimed at promoting healthy behaviours while addressing the social equity challenge.

## Supplementary Information

Below is the link to the electronic supplementary material.

(PDF 1.1 MB)

## Data Availability

The repository for the H24 library used to generate a synthetic population is available at https://github.com/eighties-cities/h24. The repository for the 5aDay agent-based model to simulation the dynamics of dietary behaviour is available at https://github.com/eighties-cities/5aday. The R computational notebook reproducing the main figures and tables of the article is available at https://eighties-cities.github.io/Paper/Notebook_R_fig_tables/Companion_h24_5aDay.html and the corresponding repository with data and code at https://github.com/eighties-cities/Paper/tree/main/Notebook_R_fig_tables.

## Footnotes

1 This idea that the ‘local’ scale (i.e. residential neighbourhoods) is the ‘only’ meaningful unit of interest in neighborhoods effects has been referred to as the ‘Local Trap’ [21].

2 Duncan index of dissimilarity is a measure of the evenness with which two groups are distributed across the spatial units that make up a city. A group is evenly distributed when each spatial unit has the same percentage of group members as the total population. Duncan Index of Dissimilarity can be interpreted as the percentage of group members who would have to move to different spatial unit in order to produce a distribution that matches that of the city. Values range from 0 (no segregation) to 1 (maximum segregation).

3 https://mobiliscope.cnrs.fr/en.

4 https://github.com/eighties-cities/h24.

5 https://eighties-cities.github.io/h24/.

6 https://github.com/eighties-cities/5aday.

7 https://eighties-cities.github.io/Paper/Notebook_R_fig_tables/Companion_h24_5aDay.html.

8 This proportion is obtained by ventilating the population of the two lower age groups into ages following a uniform distribution, and by adding the simulation duration of 6 years to this age. 17% is the proportion of agents whose estimated updated age had crossed one of the age group thresholds (30 and 60).

## Abbreviations

ABM: Agent-based model
COVID: Coronavirus Disease
CPU: Central Processing Unit
CS: Constraint Strength
EGI: European Grid Infrastructure
EGT: Enquête Globale Transport
EII: Education Inequality Index
GPS: Global Positioning System
HDC: Healthy Diet Reward
IC: Inertia Coefficient
INSEE: Institut National de la Statistique et des Études Économiques - National Institute of Statistics and Economic Studies
IRIS: Ilots Regroupés pour l’Information Statistique - aggregated units for statistical information
KISS: Keep it Simple, Stupid
KIDS: Keep it Descriptive, Stupid
MPS: Maximum Probability to Switch
NSGA2: Non-dominated Sorting Genetic Algorithm
ODE: Ordinary Differential Equations
OSE: Origin Space Exploration
PNNS: Programme National Nutrition Santé - National Nutrition and Health Program
