# Supplementary material for: An agent-based model to investigate the effects of urban segregation around the clock on inequalities in health behaviour
Source: EPJ Data Sci. 2025 Dec 11;15(1):5. doi: 10.1140/epjds/s13688-025-00603-4 (PMC12804204; doi:10.1140/epjds/s13688-025-00603-4)
Supplement: Supplementary file 1 — (PDF 1.1 MB) [file 13688_2025_603_MOESM1_ESM.pdf]

Supplementary Material.
An agent-based model to investigate the effects of urban segregation around the clock on inequalities in health behaviour.

Clémentine Cottineau-Mugadza[1], Julien Perret[2], Romain Reuillon[3], Sébastien Rey-Coyrehourcq[4], and Julie Vallée[5,*]

[1]UMR 8097 Centre Maurice Halbwachs, CNRS, Paris (FR) / Delft University of Technology ABE, Delft (NL) / c.cottineau@tudelft.nl

[2]Univ Gustave Eiffel, ENSG, IGN, LASTIG, Champs-sur-Marne (FR) / julien.perret@ign.fr

[3]Institut des Systèmes Complexes Paris Ile-de-France (FR) / UMR 8504 Géographie-cités, CNRS, Paris (FR) / romain.reuillon@iscpif.fr

[4]UMR 6266 IDEES, Université de Rouen (FR) / sebastien.rey-coyrehourcq@univ-rouen.fr

[5]UMR 8504 Géographie-cités, CNRS, Paris (FR) / UMR 5193 LISST, CNRS, Toulouse (FR) / corresponding author: julie.vallee@cnrs.fr

[*]Authors are listed in alphabetical order

1 Segregation around the clock for gender, age and educational groups in the Paris region in 2010

Duncan's segregation index measures the unevenness of a group distribution compared to the rest of the population. Values range from 0 (no segregation) to 1 (maximum segregation).

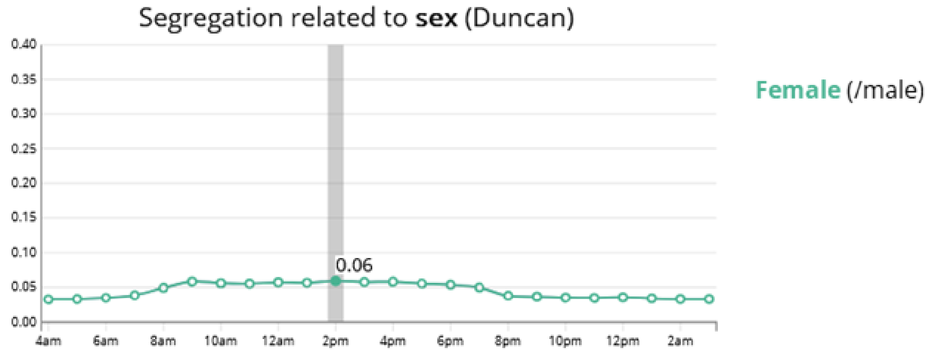

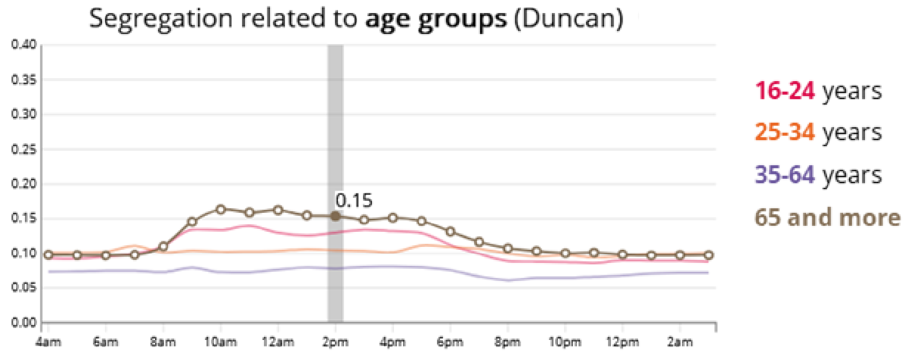

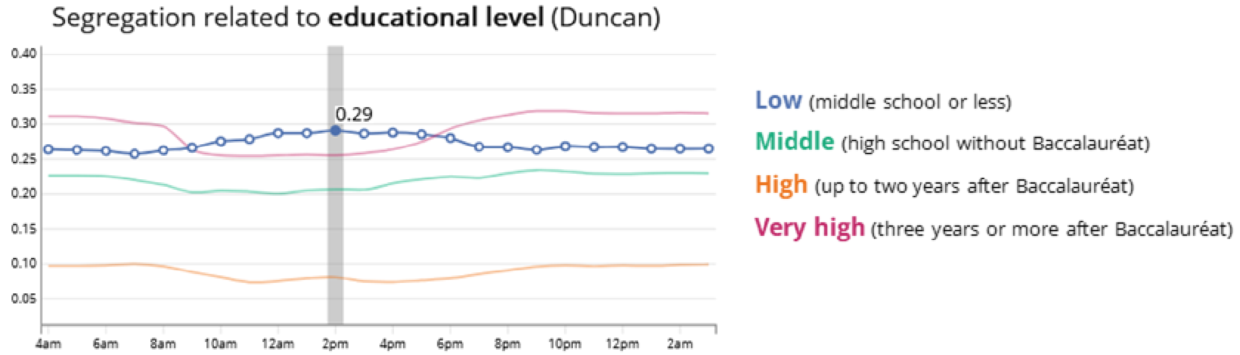

Source : Screenshots from Mobiliscope (v4.3) www.mobiliscope.cnrs.fr (Vallée et al., 2024) ; 2010 origin-destination survey (EGT)

2 Distribution of synthetic population in the Paris region across the 18 sociodemographic categories and moves between 'night, 'day', and 'evening' cells (H24 library)

**Sex**	**Age**	**Education**	**nb agents**	**% of agents**	**% with a 'day' cell which differs from the 'night' cell**	**% with an 'evening' cell which differs from the 'night' cell**
Male	15-29 yrs.	low	493,871	5.6%	86%	70%
Male	15-29 yrs.	middle	496,964	5.7%	83%	62%
Male	15-29 yrs.	high	199,476	2.3%	90%	67%
Male	30-59 yrs.	low	1,077,670	12.3%	77%	47%
Male	30-59 yrs.	middle	662,407	7.6%	82%	54%
Male	30-59 yrs.	high	658,465	7.5%	85%	44%
Male	60-75 yrs.	low	299,094	3.4%	71%	65%
Male	60-75 yrs.	middle	182,988	2.1%	83%	79%
Male	60-75 yrs.	high	187,662	2.1%	64%	52%
Female	15-29 yrs.	low	501,631	5.7%	84%	69%
Female	15-29 yrs.	middle	547,970	6.2%	83%	58%
Female	15-29 yrs.	high	193,543	2.2%	84%	58%
Female	30-59 yrs.	low	1,107,531	12.6%	70%	45%
Female	30-59 yrs.	middle	813,463	9.3%	79%	49%
Female	30-59 yrs.	high	596,958	6.8%	77%	40%
Female	60-75 yrs.	low	321,202	3.7%	65%	58%
Female	60-75 yrs.	middle	242,236	2.8%	75%	70%
Female	60-75 yrs.	high	185,767	2.1%	64%	55%
		Total	*8,768,898*	*100%*	*78%*	*54%*

Source: 2012 Census (INSEE) and 2010 origin-destination survey (EGT)

3 Spatial segregation of synthetic population (H24 Library) in 'night', 'day' and 'evening' cells in the Paris region

Duncan index of Dissimilarity (D) for each sociodemographic category. It measures the unevenness of a group distribution compared to the rest of the population. Values range from 0 (no segregation) to 1 (maximum segregation).

Sex	Age	Education	Duncan (D) 'night' cell	Duncan (D) 'day' cell	Duncan (D) 'evening' cell
Male	15-29 yrs.	low	0.152	0.549	0.548
Male	15-29 yrs.	middle	0.082	0.442	0.423
Male	15-29 yrs.	high	0.257	0.57	0.55
Male	30-59 yrs.	low	0.182	0.394	0.372
Male	30-59 yrs.	middle	0.091	0.391	0.345
Male	30-59 yrs.	high	0.274	0.437	0.386
Male	60-75 yrs.	low	0.184	0.564	0.553
Male	60-75 yrs.	middle	0.121	0.665	0.698
Male	60-75 yrs.	high	0.284	0.528	0.56
Female	15-29 yrs.	low	0.138	0.558	0.559
Female	15-29 yrs.	middle	0.088	0.445	0.396
Female	15-29 yrs.	high	0.275	0.492	0.493
Female	30-59 yrs.	low	0.16	0.371	0.362
Female	30-59 yrs.	middle	0.086	0.364	0.32
Female	30-59 yrs.	high	0.263	0.375	0.373
Female	60-75 yrs.	low	0.15	0.501	0.481
Female	60-75 yrs.	middle	0.131	0.573	0.592
Female	60-75 yrs.	high	0.295	0.57	0.605
Male			0.033	0.23	0.159
Female			0.033	0.23	0.159
15-29 yrs.			0.076	0.297	0.262
30-59 yrs.			0.052	0.28	0.224
60-75 yrs.			0.095	0.37	0.345
low			0.241	0.349	0.361
middle			0.067	0.252	0.236
high			0.322	0.358	0.404

Moran's spatial-autocorrelation index (M) for each sociodemographic category. It measures spatial-autocorrelation. It is computed on the proportion of agents of a specified group with equal weight given to each occupied neighbouring cell distribution. Values ranges from -1 (when cells that are spatially close to each other tend to have dissimilar social composition - negative autocorrelation) to 1 (when cells that are spatially close to each other tend to have similar social composition - positive autocorrelation. A value close to zero expresses no spatial structure.

Sex	Age	Education	Moran (M) 'night' cell	Moran (M) 'day' cell	Moran (M) 'evening' cell
Male	15-29 yrs.	low	0.022	0.182	0.125
Male	15-29 yrs.	middle	0.035	0.197	0.193
Male	15-29 yrs.	high	0.069	0.184	0.113
Male	30-59 yrs.	low	0.1	0.085	0.088
Male	30-59 yrs.	middle	0.003	0.06	0.078
Male	30-59 yrs.	high	0.198	0.062	0.074
Male	60-75 yrs.	low	0.027	0.187	0.196
Male	60-75 yrs.	middle	0.013	0.331	0.351
Male	60-75 yrs.	high	0.068	0.11	0.107
Female	15-29 yrs.	low	0.02	0.189	0.192
Female	15-29 yrs.	middle	0.04	0.126	0.112
Female	15-29 yrs.	high	0.031	0.104	0.077
Female	30-59 yrs.	low	0.066	0.06	0.072
Female	30-59 yrs.	middle	0.01	0.036	0.057
Female	30-59 yrs.	high	0.11	0.055	0.054
Female	60-75 yrs.	low	0.023	0.086	0.095
Female	60-75 yrs.	middle	0.029	0.325	0.352
Female	60-75 yrs.	high	0.05	0.225	0.232
Male			0.018	0.15	0.148
Female			0.018	0.15	0.148
15-29 yrs.			0.076	0.175	0.145
30-59 yrs.			0.026	0.239	0.262
60-75 yrs.			0.068	0.279	0.306
low			0.303	0.226	0.201
middle			0.071	0.201	0.193
high			0.367	0.172	0.166

4 Measure of rank-ordered inequalities

We also compute the corrected concentration index E_t proposed by Erreygers (2009) to analyse rank-ordered inequalities of health variables.

$$E_t = \frac{8}{n^2} * \sum_{edu=1}^{3} z_{edu} * H_{edu} \tag{1}$$

with H_{edu} the number of people eating 5-a-day in the given education category.

$$z_{edu} = \frac{n+1}{2} - \lambda_{edu} \tag{2}$$

with λ_{edu} the median rank of the people of the given education category. The concentration index H_{edu} varies between -1 (the healthy diet is for less educated people) and 1 (the healthy diet is for more educated people). 0 represents the case where people of all education categories have no difference in terms of dietary behaviour.

From observed data from the two Health and Nutrition Barometer Surveys (with population distribution by age and sex corresponding to Paris region from 2012 census database), E_t was found to increase from 0.0175 in 2002 to 0.0583 in 2008.

From 10 000 replications of the agent-based model, rank-ordered index E_t increases from 0.0174 (at initialisation) to a median of 0.0444 as at the end of the simulation.

Distribution of simulated values of rank-ordered inequality index (E_t) for the five space-time scenarios

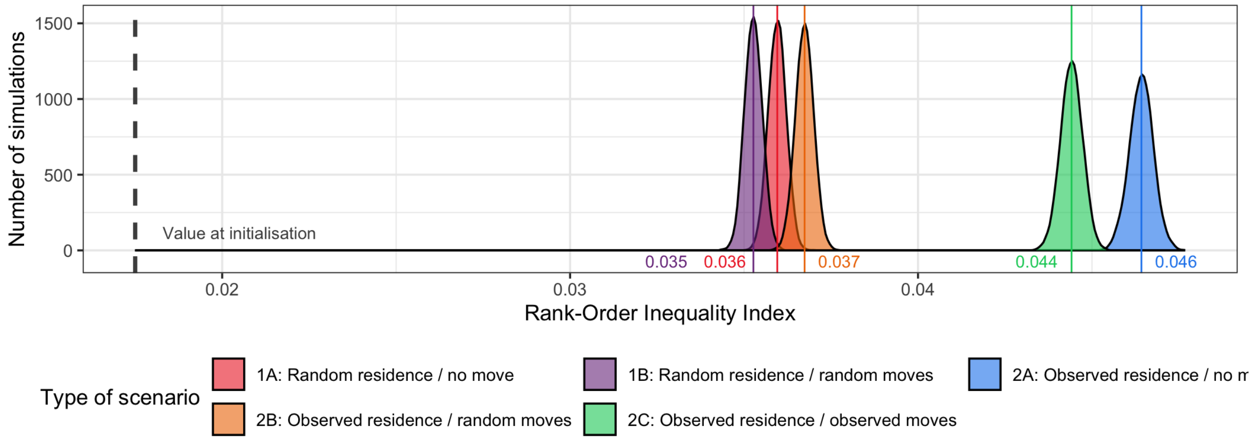

5 Dietary attributes for agents at initialisation across the 18 sociodemographic categories ('5aDay' model)

Sex	Age	Education	nb agents	% of agents	agents eating fruit and vegetable 5-a-day (%)	average of opinion among healthy agents	average of opinion among unhealthy agents
Male	15-29 yrs.	low	493,871	5.6%	1.9%	0.50	0.40
Male	15-29 yrs.	middle	496,964	5.7%	3.1%	0.37	0.42
Male	15-29 yrs.	high	199,476	2.3%	3.5%	0.70	0.42
Male	30-59 yrs.	low	1,077,670	12.3%	5.0%	0.52	0.40
Male	30-59 yrs.	middle	662,407	7.6%	7.4%	0.50	0.44
Male	30-59 yrs.	high	658,465	7.5%	5.9%	0.64	0.46
Male	60-75 yrs.	low	299,094	3.4%	14.6%	0.47	0.43
Male	60-75 yrs.	middle	182,988	2.1%	3.3%	0.50	0.43
Male	60-75 yrs.	high	187,662	2.1%	17.7%	0.50	0.38
Female	15-29 yrs.	low	501,631	5.7%	3.0%	0.40	0.44
Female	15-29 yrs.	middle	547,970	6.2%	5.0%	0.60	0.47
Female	15-29 yrs.	high	193,543	2.2%	7.0%	0.56	0.49
Female	30-59 yrs.	low	1,107,531	12.6%	13.5%	0.57	0.48
Female	30-59 yrs.	middle	813,463	9.3%	14.6%	0.63	0.48
Female	30-59 yrs.	high	596,958	6.8%	14.5%	0.60	0.52
Female	60-75 yrs.	low	321,202	3.7%	20.0%	0.52	0.50
Female	60-75 yrs.	middle	242,236	2.8%	26.0%	0.57	0.52
Female	60-75 yrs.	high	185,767	2.1%	23.7%	0.60	0.48
		Total	*8,768,898*	*100%*	*9.6%*	*0.54*	*0.45*

6 Exploration of Pareto Front

Distribution of parameter values corresponding to optimal parameter sets on the Pareto front

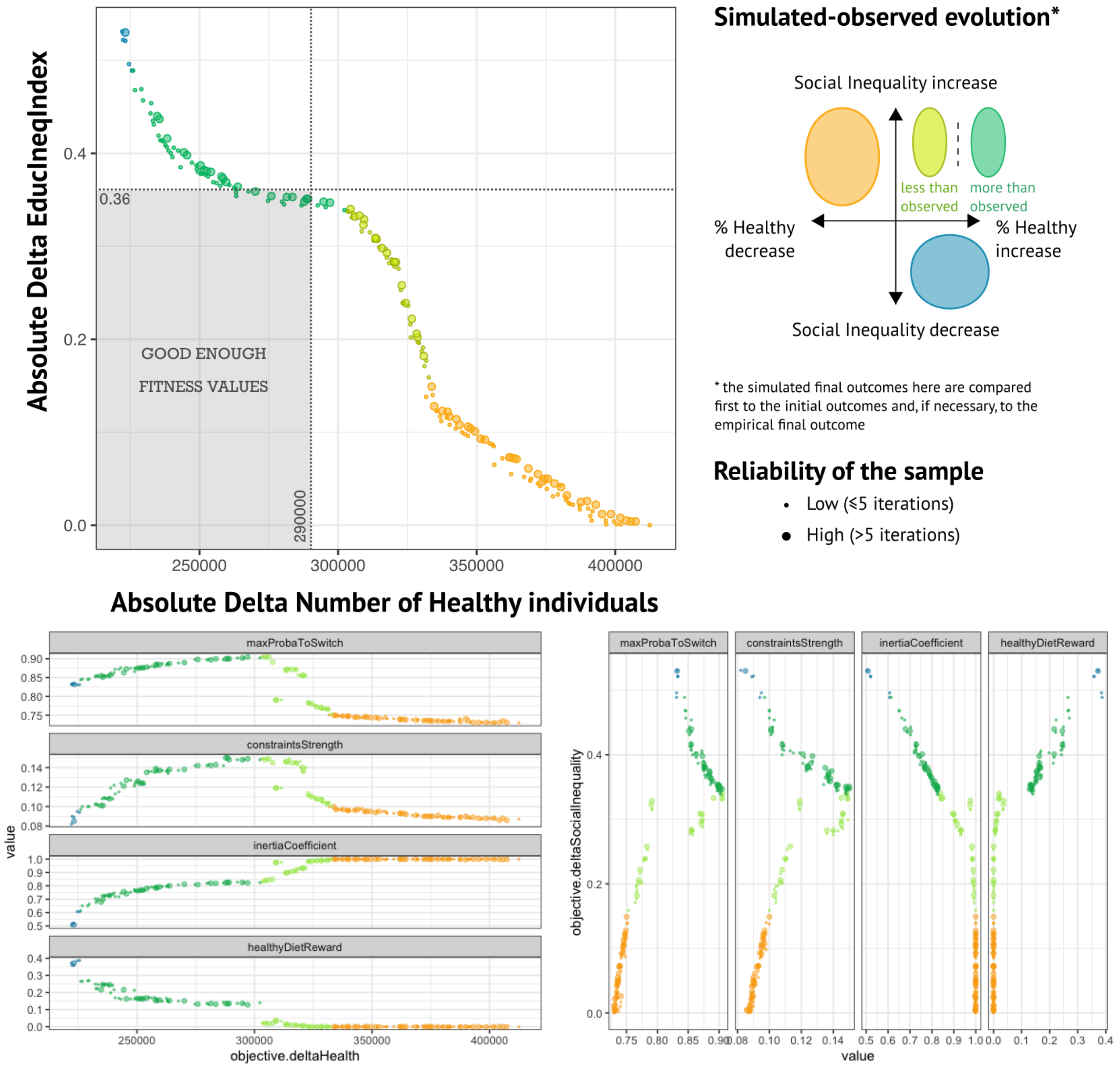

7 Origin Space Exploration (OSE) method

A method to discover the subpart of the parameter space which leads to acceptable outputs

Origin Space Exploration (OSE) is a novel exploration method (it has not been published yet). OSE is mostly similar to an NSGA2 optimisation, except that once a zone of the parameter space is found to be containing good-enough fitness values, they are archived and the part of the input space containing the solution is excluded from the search space by rejection: : if the sampler generates a solution inside one of the known zone this sample is rejected and another one is drawn.

In addition to the multi-objective fitness function and to the input exploration space definition that is requires for NSGA2 to work, OSE requires the definition of an inequality defined on the fitness function as well as a partition of the input space.

The inequality is on the fitness function makes it possible to define what is a good-enough fitness. For instance let say that the optimisation minimize three objective $o1, o2, o3$, lets define 3 boundaries $b1, b2, b3$. A good enough solution is defined as a set of inputs for which the matching outputs satisfy the predicate $o1 < b1 \ \& \ o2 < b2 \ \& \ o3 < b3$.

Given the set of satisfying solutions already found $\{S\}$ at a given point in the algorithm the parts of the input space containing these solution is excluded from the search space. To achieve this exclusion OSE uses a sampling method with rejection based on the partitioning of the space provided to the method. OSE draw the candidate offspring genomes in the same manner as NSGA2 but the genomes drawn in subpart of the space (as defined by the partitioning) that contain a genome existing $\{S\}$ are rejected an an new genome is drawn.

We compute the full subset of the parameter space meeting *good-enough fitness values* previously defined. In the present paper, we have defined our *good-enough fitness values* from $\Delta_{Health} <$ $290,000$ and $\Delta_{EII} < 0.36$. The partitioning of input space is a split in hyper-cubic zones of side 0.1. Each time a parameter set producing *good-enough fitness values* was found, the entire hyper-cubes containing this point was black-listed and the optimisation continued in the remaining of the space.

Dynamic resampling - Handling Stochasticity in OSE

In this paper we applied OSE to explore a stochastic model. To handle the stochasticity, we combined this method with used a dynamic resampling method already described in the Chapter 3 of Pumain and Reuillon (2017):

> A problem when using genetic algorithms to calibrate simulation models is that some of them don't cope well with stochasticity. This is especially the case for algorithm of type $\mu + \lambda$ (such as NSGA2) which preserve best solutions between the generations. In that kind of optimisation the value of a solution is only estimated and not computed exactly. They can therefore be overvalued or undervalued (the quality of a solution is estimated with a significantly greater or a lower value than the one that would have been estimated given an infinite number of replications). Undervalued solutions are not very problematic for $\mu + \lambda$ genetic algorithms, they might be discarded instead of being kept, but the algorithm has a chance to retry a very similar solution later on. On the contrary the overvalued solution are very problematic for genetic algorithms, since the genetic algorithm might keep overvalued solution in the population of good solutions (because they have falsely been evaluated as good solutions) and generates new offspring solutions

from them. This behaviour can greatly slow down the convergence of the calibration algorithm and even make it converge toward set of parameters producing very unstable output dynamics which are very likely to produce false positive good solutions.

To reduce the influence of the fitness fluctuation, the most commonly used approach is called "resampling". It consists in running several replications for each fitness evaluation. The computed quality for a set of parameter is then an estimation given a finite number of replications of the fitness computation. However to limit the computation time taken to evaluate the quality of a single set of parameters during the calibration process, the number of replications is generally limited to a level which constitute a compromise between the computation time taken to evaluate one set of parameter and a acceptable level of noise for the quality. Any number of replications even very high, still implies that some solutions are overvalued with a non negligible probability given that the fitness function is evaluated millions of times.

Other methods have been developed to optimise stochastic functions using genetic algorithm. Some of them are based on using the history of the genetic algorithm to estimate probability distribution of the fitness (Sano and Kita (2002)), other are based on the differences between the parents and the offspring (Tanooka et al. (1999)) and others propose to use a partial order base on statistical tests (Rudolph (2001))... Even if these methods seems statistically sounded they complicate significantly the optimisation algorithm, they are often based some assumptions that are hard or impossible to verify (such as the invariance of the noise distribution over the fitness space) and they add parameters to the algorithm that are hard to tune.

To overcome these limitations we have developed an auto-adaptive strategy to handle stochastic fitness functions in NSGA2. It is loosely related to the idea of resampling, for which only the best solutions are more precisely evaluated (presented in Branke (1998)). In our method, called "stochastic resampling" we propose to evaluate the individual with only 1 replication and then to resample the individuals of the population with a fixed probability at each generation of the evolutionary algorithm. For instance, at each generation 90% of the individual offspring genomes are new genomes and 10% of the offspring genomes are already evaluated genomes randomly taken in the current population for which the algorithm computes one additional replication. The replications of each individual are stored in a vector of replications. The fitness of an individual is computed using (for instance) the median of each objective stored in the replication vector. The intuition is that the in $\mu + \lambda$ genetic algorithms, best individuals survive several generations and therefore are the most likely to be resampled given that each individual has a fixed chance of being resampled at each generation. However, this fixed probability of resampling is not sufficient by itself to get an auto-adaptive algorithm. With this mechanism alone, well evaluated solutions are very likely to be replaced by overvalued ones (new solution with a few "lucky" replications). To compensate this bias, we add a technical objectives in NSGA2 in order to maximise the number of samples of a solution to the multi-objective optimisation problem. Therefore the number of replications is taken into account in the Pareto compromise elitism of NSGA2: solutions with many replication are kept even if some solution are better on the other objectives but have been evaluated with less replications. By doing so, we let the multi-objective optimisation algorithm handle the compromise between the quality of the solutions and their robustness. This method add only two new parameters: 1/ the probability

of resampling an individual at each generation 2/ the max number of samples for an individual to limit the memory used to store an individual. We propose to store the sample in a FIFO with a fixed size, therefore new samples are always taken into account even if the maximum number of replication has been reach for a given individual. This method has been implement in the library for evolutionary computing: MGO[1] and has not been published yet.

[1]https://github.com/openmole/mgo

8 Evolution in simulated values ('5aDay' model) over the six series of three time slices

Evolution in simulated **proportion of healthy agents** (part A) and in simulated values of **Social inequality index** EII (part B).

From 100 replications of '5aDay' model (for scenarios 2C and 1B).

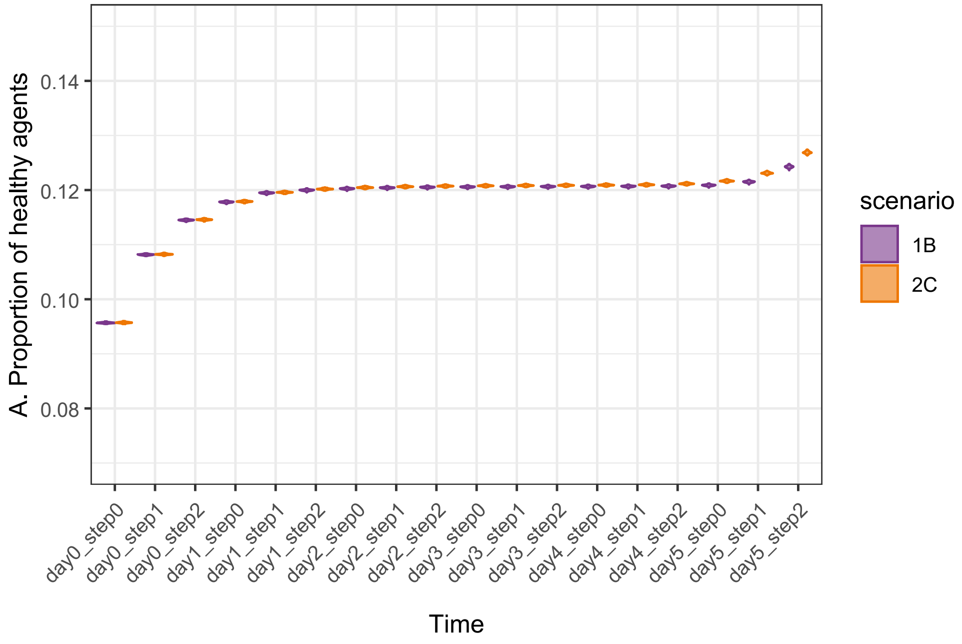

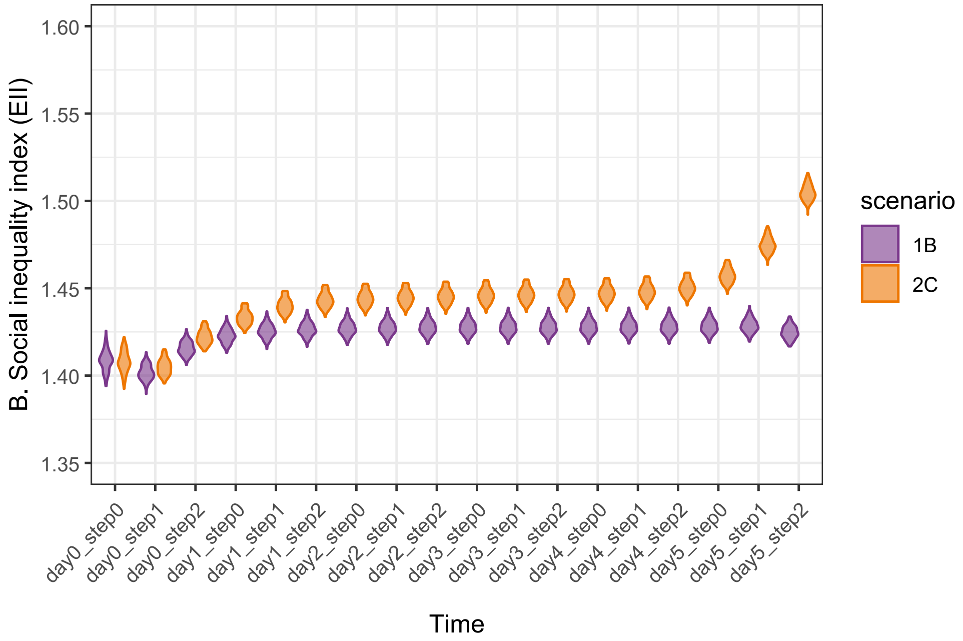

Evolution in simulated **proportion of healthy agents** (part C) and simulated **opinion** (part D) across the 18 sociodemographic categories.

From 1 replication of '5aDay' model (for scenario 2C).

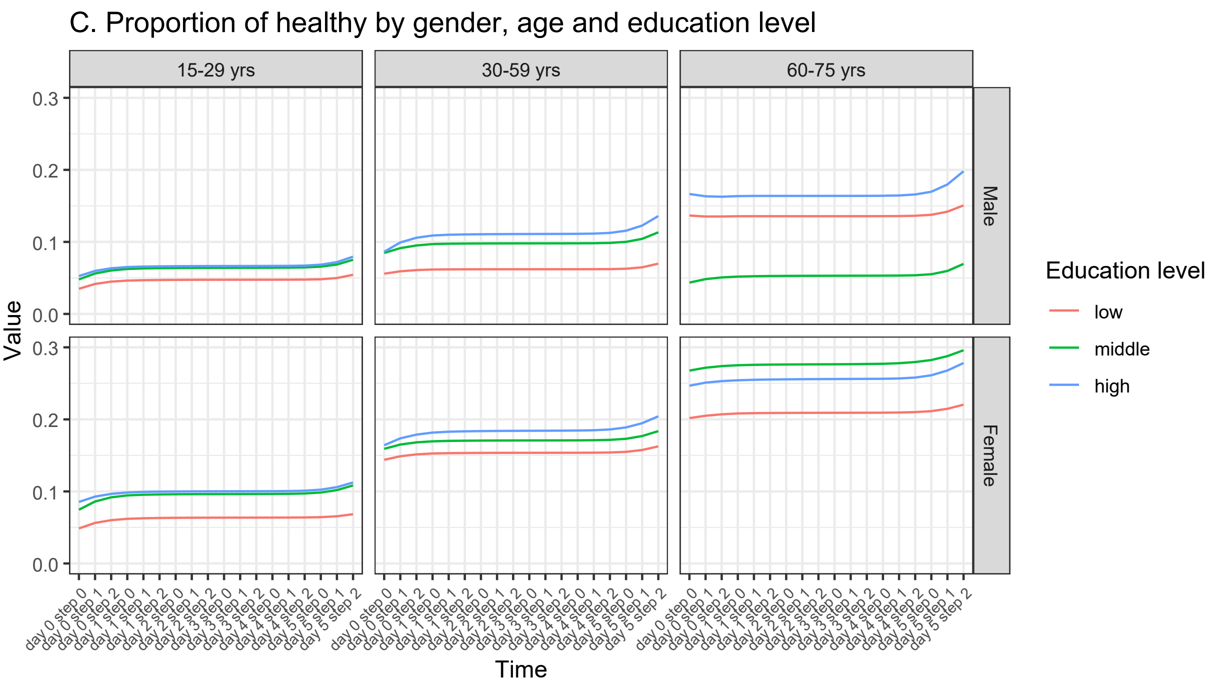

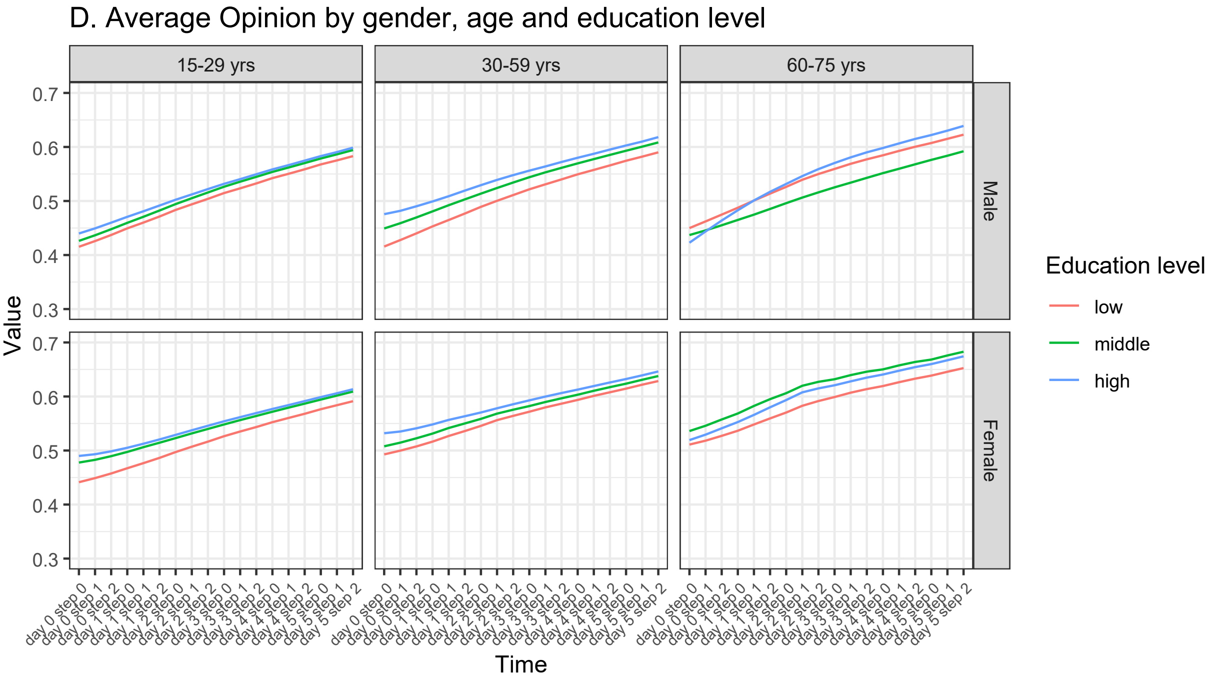

9 References

Branke, J.
1998. *Parallel Problem Solving from Nature — PPSN V: 5th International Conference Amsterdam, The Netherlands September 27–30, 1998 Proceedings*, chapter Creating robust solutions by means of evolutionary algorithms, Pp. 119–128. Berlin, Heidelberg: Springer Berlin Heidelberg.

Erreygers, G.
2009. Correcting the concentration index. *Journal of health economics*, 28(2):504–515.

Pumain, D. and R. Reuillon
2017. *Urban Dynamics and Simulation Models*, Lecture Notes in Morphogenesis. Springer International Publishing.

Rudolph, G.
2001. A partial order approach to noisy fitness functions. In *Congress on Evolutionary Computation, Seoul, Korea*, Pp. 318–325. Press.

Sano, Y. and H. Kita
2002. Optimization of noisy fitness functions by means of genetic algorithms using history of search with test of estimation. In *Evolutionary Computation, 2002. CEC '02. Proceedings of the 2002 Congress on*, volume 1, Pp. 360–365.

Tanooka, K., H. Tamaki, S. Abe, and S. Kitamura
1999. A continuous age model of genetic algorithms applicable to optimization problems with uncertainties. In *Systems, Man, and Cybernetics, 1999. IEEE SMC '99 Conference Proceedings. 1999 IEEE International Conference on*, volume 1, Pp. 637–642 vol.1.

Vallée, J., A. Douet, G. Le Roux, H. Commenges, C. Lecomte, and E. Villard
2024. Mobiliscope, an open platform to explore cities and social mix around the clock. www.mobiliscope.cnrs.fr.
